# Label-free imaging of red blood cells and oxygenation with color third-order sum-frequency generation microscopy

**DOI:** 10.1038/s41377-022-01064-4

**Published:** 2023-01-26

**Authors:** Júlia Ferrer Ortas, Pierre Mahou, Sophie Escot, Chiara Stringari, Nicolas B. David, Laure Bally-Cuif, Nicolas Dray, Michel Négrerie, Willy Supatto, Emmanuel Beaurepaire

**Affiliations:** 1 Laboratory for Optics and Biosciences, CNRS, INSERM, École polytechnique, IP Paris, 91128 Palaiseau, France; 2 Zebrafish Neurogenetics Unit, team supported by Ligue Nationale contre le Cancer, Institut Pasteur, CNRS, 75015 Paris, France

**Keywords:** Multiphoton microscopy, Nonlinear optics

## Abstract

Mapping red blood cells (RBCs) flow and oxygenation is of key importance for analyzing brain and tissue physiology. Current microscopy methods are limited either in sensitivity or in spatio-temporal resolution. In this work, we introduce a novel approach based on label-free third-order sum-frequency generation (TSFG) and third-harmonic generation (THG) contrasts. First, we propose a novel experimental scheme for color TSFG microscopy, which provides simultaneous measurements at several wavelengths encompassing the Soret absorption band of hemoglobin. We show that there is a strong three-photon (3P) resonance related to the Soret band of hemoglobin in THG and TSFG signals from zebrafish and human RBCs, and that this resonance is sensitive to RBC oxygenation state. We demonstrate that our color TSFG implementation enables specific detection of flowing RBCs in zebrafish embryos and is sensitive to RBC oxygenation dynamics with single-cell resolution and microsecond pixel times. Moreover, it can be implemented on a 3P microscope and provides label-free RBC-specific contrast at depths exceeding 600 µm in live adult zebrafish brain. Our results establish a new multiphoton contrast extending the palette of deep-tissue microscopy.

## Abstract (web summary)

Color TSFG microscopy is a novel approach for label-free imaging of red blood cells and oxygenation in situ.

## Graphical abstract

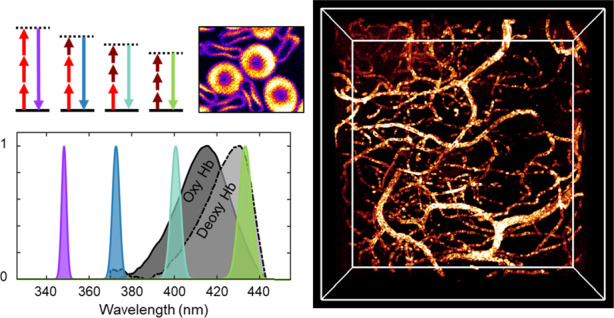

## Introduction

Cells and tissues rely on red blood cells (RBCs) circulation for oxygen supply. Analyzing tissue metabolism during normal and pathological activity requires quantifying hemodynamics and blood oxygenation at high resolution in situ. Two-photon (2P) microscopy is a reference technique for measuring RBC microcirculation^1–3^ and blood oxygenation using exogenous phosphorescent probes^4–7^ and is increasingly used to study brain oxygenation and physiology. Despite their performances, 2P-based methods face some limitations: oxygenation measurements based on phosphorescence usually involve pixel times in the tens-of-millisecond range^7^ and probe the blood plasma rather than probing the RBCs directly; fluorescence-based microcirculation measurements rely on blood labeling or suffer from relatively weak autofluorescence^8^; in-depth 2P microscopy is ultimately limited by out-of-focus background^9^. On a partly related topic, the recent advent of high-power optical parametric amplifiers (OPA) and sources at MHz repetition rates in the 1.3 and 1.7 μm ranges^10–12^ has opened novel perspectives for deep-tissue microscopy using three-photon (P) excitation, such as mouse brain imaging at depths exceeding one millimeter^10,11,13,14^. A side benefit of these novel laser technologies is that they also enable efficient label-free imaging based on third-harmonic generation (THG). THG^15,16^ is a third-order multiphoton imaging modality highlighting interfaces and optical heterogeneity in cells and tissues with applications ranging from cell and developmental biology to neuroscience^17–24^. THG microscopy is easily combined with 3P fluorescence microscopy for deep-tissue imaging combining both contrast modalities. In live imaging of mice and fish brains, THG has been shown to predominantly reveal blood vessels, myelin-rich areas, and skull interfaces^10–14,19,25–28^. Although THG contrast has so far principally been used for structural imaging and as a spatial landmark, it is anticipated that some degree of chemical specificity for absorbing objects could be obtained by exploiting the fact that the third-order nonlinear susceptibility *χ*^(3)^ (3*ω*; *ω*, *ω*, *ω*) is altered when frequency *ω*, 2*ω* or 3*ω* matches an electronic resonance in the sample. Following this line of inquiry, a handful of pioneering studies have revealed the existence of wavelength-dependent resonances in THG from hemoglobin and other absorbers^29–34^. In addition, it is well known that the linear absorption spectra of hemoglobin in its oxygenated and deoxygenated forms are different, particularly near the Soret absorption band in the 415–430 nm range.

A spectroscopic version of THG microscopy could therefore be of particular relevance for probing the absorption properties of non-fluorescent objects. In particular, it could provide specific contrast from RBCs and potentially report on their oxygenation state in vivo in a label-free manner. The development of such a functional imaging approach however requires (i) to establish and characterize the wavelength dependence of THG signals from RBCs, and (ii) to implement an efficient spectral imaging scheme compatible with microscopy of moving objects such as flowing RBCs.

In this work, we introduce a novel experimental scheme for *χ*^(3)^ spectroscopic imaging based on the simultaneous measurement of THG and third-order sum frequency generation (TSFG) signals at several emission wavelengths encompassing the Soret absorption band of hemoglobin. In turn, we demonstrate that this approach provides chemically specific *χ*^(3)^ contrast based on electronic resonance in imaging experiments of isolated red blood cells, zebrafish embryos, and live adult zebrafish brain. We also demonstrate that our method can be used to measure oxygenation dynamics in vivo.

## Results

### One-shot multicolor TSFG microscopy through wavelength mixing

**Fig. 1:**
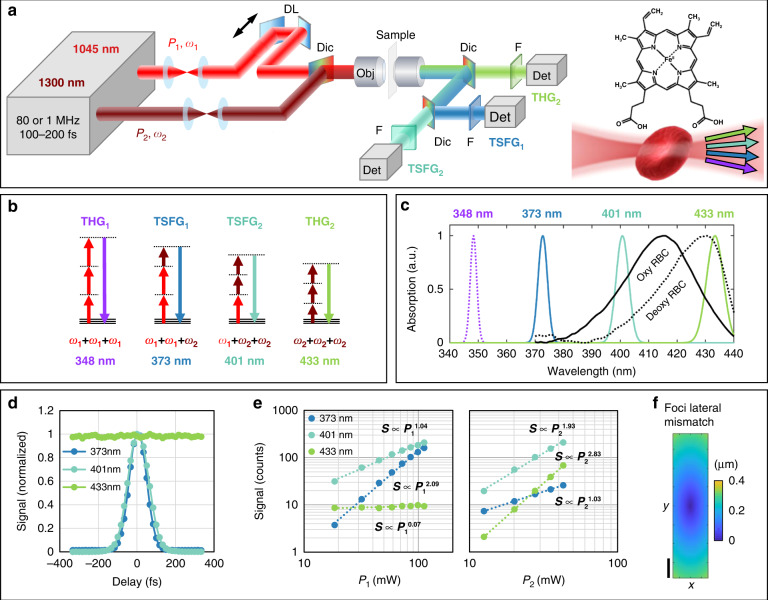
Principles of color TSFG microscopy for blood imaging. **a** Experimental setup. DL: motorized optical delay line. Dic: dichroic mirror. Obj: objective lens. F: spectral bandpass filter. Det: detector. **b** Energy diagram of the simultaneous signals processes in color TSFG microscopy, based on third-order combinations of two excitation beams at frequencies ω_1_ and ω_2_. The wavelengths indicated are those produced in the case of mixed 1045 and 1300 nm excitation. **c** Measured linear absorption spectra of zebrafish RBCs in deoxygenated (dotted line) and oxygenated (solid line) states. The wavelengths generated simultaneously by color TSFG encompass the Soret absorption band of hemoglobin, enabling spectroscopic measurements. **d** Measured TSFG_1_, TSFG_2_ and THG_2_ signals from a water-glass horizontal interface as a function of the temporal delay between the two excitation pulse trains. **e** Measured TSFG_1_, TSFG_2_ and THG_2_ signals from polystyrene beads as a function of the excitation power of the pump beam (P_1_) and OPO beam (P_2_), consistent with Eq. 1. **f** Excitation beam foci lateral mismatch along the *y*-direction, reaching a maximum of 0.4 μm separation 400 µm away from the optical center. Scale bar: 100 μm. The measurement is based on data from 424 fluorescent beads distributed across the field of view

Our strategy for implementing multicolor *χ*^(3)^ contrast in a multiphoton microscope is to spatially and temporally overlap two femtosecond pulse trains in the 1045 and 1300 nm ranges, and to detect simultaneously three or four THG and TSFG signals produced by this excitation scheme, anticipating that this will be equivalent to a multicolor THG experiment (Fig. 1a). In practice, this excitation regime can easily be implemented with recent generations of short-wavelength infrared (IR) optical parametric oscillators (OPOs)^35,36^ or amplifiers (OPAs), by using a fraction of the pump beam for excitation in addition to the parametric beam. Suitable sources include the 80 MHz femtosecond OPOs commonly used for 2 P microscopy of red fluorescent proteins and for THG imaging, and the 1 MHz femtosecond OPAs increasingly used for 3P microscopy and THG^11,12^. Overlapping the pump beam at wavelength *λ*_1_ with the IR parametric pulse train at *λ*_2_ in a medium with non-zero *χ*^(3)^ produces two THG signals at *λ*_1_/3 and *λ*_2_/3, as well as two TSFG signals at 1/(2/*λ*_1_+1/*λ*_2_) and 1/(1/*λ*_1_+2/*λ*_2_) (see Fig. 1b). Assuming *λ*_1_ = 1045 nm and *λ*_2_ = 1300 nm, these four signals are simultaneously produced at ~348, 373, 401 and 433 nm, i.e. a range of wavelengths corresponding to different points around the Soret absorption band of hemoglobin (Fig. 1c). In our implementation, we directed the 373, 401, and 433 nm light to three independent detectors. The 348 nm light is poorly transmitted by standard microscope components and can only be detected with UV-coated optics. One distinctive advantage of such implementation is that it provides a one-shot spectroscopic measurement: the simultaneous detection at three wavelengths enables ratiometric imaging of moving objects such as RBCs in vivo, as we will discuss below.

Before demonstrating color TSFG microscopy for cell and tissue imaging, we provide proof of the nature of the signals through the analysis of their dependences on experimental parameters. Specifically, we analyzed contrast mechanism, signal dependence on excitation power of both beams, delay between the pulse trains, relative duration of the two pulses, and spatial overlap of the beams across the field of view.

We recorded simultaneous THG-TSFG signals from 1 μm polystyrene beads embedded in agarose (see Methods) and from horizontal water-glass interfaces, and measured the dependence of these signals on beam power and pulse delay. The signals at 373 nm (TSFG_1_) and 401 nm (TSFG_2_) were observed only when the pulses were temporally overlapped within ± 100 fs (Fig. 1d), in contrast with the THG_2_ signal at 433 nm produced by the OPO beam only. As expected, the detected TSFG_1_, TSFG_2_, and THG_2_ signals were observed to depend on beam average powers as: $S_{{{{\mathrm{THG}}}}_2} \propto P_2^3$, $S_{{{{\mathrm{TSFG}}}}_1} \propto P_1^2P_2$, $S_{{{{\mathrm{TSFG}}}}_2} \propto P_1P_2^2$ (Fig. 1e). The fact that these three signals exhibit different dependence on beam powers and on pulse delay provides an experimental means to balance their relative levels, as in the case of trichromatic 2P excitation with wavelength mixing^37^.

We estimated the resolution of the two imaging modalities by extracting lateral and axial profiles from point objects located at the center of the field of view and found similar values for all signals, namely an axial spread of 1.8 ± 0.2 µm FWHM and a lateral spread of 0.5 ± 0.1 µm FWHM for 0.5 µm beads. We then compared the contrast mechanisms of THG and TSFG by extracting axial profiles through horizontal water-glass interfaces. We observed similar behaviors for both modalities (Fig. S1): all signals reach a maximum when the interface is in focus, and decrease to zero when the focus is either inside the water or the glass coverslip. This observation indicates that the phase-matching condition and resulting contrast mechanism are similar for THG and TSFG in our excitation conditions. Due to the limited excitation wavelength range involved and the similar NAs of the two excitation beams, TSFG here behaves as a THG signal at a different excitation wavelength.

We then analyzed the dependence of TSFG signals on relative pulse durations of the two beams. Assuming a Gaussian temporal profile for the pulses $I\left( t \right) = \left( {I_0/\sqrt {2\pi } } \right)\left( {T/\tau } \right)\exp \left\{ { - t^2/2\tau ^2} \right\}$ with maximum intensity *I*_0_, repetition rate 1/*T*, and pulse duration *τ*, the THG and TSFG signals *S*, should exhibit the following dependence on the pump and OPO beam average powers, *P*_1_ and *P*_2_, and pulse durations, *τ*_1_ and *τ*_2_, respectively (see^38^ and Supplementary text):

1 $$S_{{{{\mathrm{THG}}}}_1} \propto \left( {T/\tau _1} \right)^2\left\langle {P_1} \right\rangle ^3 \quad ; \quad S_{{{{\mathrm{THG}}}}_2} \propto \left( {T/\tau _2} \right)^2\left\langle {P_2} \right\rangle ^3$$

2 $$S_{{{{\mathrm{TSFG}}}}_1} \propto \frac{{9\sqrt 3 \,T^2}}{{\tau _1\tau _2\sqrt {(\tau _1/\tau _2)^2 + 2} }}\left\langle {P_1} \right\rangle ^2\left\langle {P_2} \right\rangle \quad ; \quad S_{{{{\mathrm{TSFG}}}}_2} \propto \frac{{9\sqrt 3 \,T^2}}{{\tau _1\tau _2\sqrt {(\tau _2/\tau _1)^2 + 2} }}\left\langle {P_1} \right\rangle \left\langle {P_2} \right\rangle ^2$$

where Eqs. (1) and (2) assume a single-beam and dual-beam process, respectively.

We verified experimentally that the dependence of TSFG signals on relative pulse durations is properly predicted by Eq. 2. Details on these experiments are given in the Supplementary Text and Fig. S2. In short, TSFG signals are maximized when the pulse durations of the excitation beams are shortest and equal.

Finally, we analyzed the effect of foci mismatch across the field of view on TSFG signals (see Supplementary Text and Fig. S3). We experimentally observed that the TSFG/THG signals ratio decreased by ~30 ± 10 % 350 µm away from the center of the field of view (Fig. S3c). Consistent with our previous reports^12,39^, we measured that the lateral and axial mismatch between the excitation foci increases with the distance from the optical center due to chromatic aberration in the excitation path (Fig. 1f and Fig. S3b). We can attribute the TSFG/THG ratio decrease away from the optical center principally to these effects (Fig. S3). To ensure that this drop is <20 %, in the subsequent imaging experiments we restrain our quantitative measurements to a field of view of 500 µm in diameter.

### Resonances in THG spectra

THG imaging contrast is strongly influenced by wavelength-scale sample heterogeneity, which has been extensively studied^15,40–43^. In addition to this dependence on sample microstructure, it has also been shown that biological THG can be enhanced by the presence of strong absorbers such as porphyrin-based pigments. Early studies of THG microscopy reported contrast in chloroplasts^44^, cardiomyocytes^45^, hematoxylin^30,46^, hemozoin^32^, RBCs and hemoglobin^29,31,47^. Recent works have used THG in combination with second-harmonic generation and/or fluorescence imaging in murine muscles^48^, to monitor erythrocytes flow in vessels^49^, and to extract hemodynamic parameters^50,51^ 1 mm deep inside the mouse cortex or in human volunteers’ capillaries^52^. Finally, a few studies characterized THG signals near glass-hemoglobin interfaces, and identified the presence of resonances at wavelengths corresponding to 2P or 3P absorption in the Soret band^29,31^. This phenomenon could be exploited as a contrast enhancement mechanism during THG imaging of erythrocytes. However, it has not been directly characterized in RBCs in the 1000–1300 nm wavelength range. In this section, we will briefly recall the rationale for the presence of such resonances, and present a spectral characterization of THG from RBCs in oxygenated and deoxygenated states.

Resonant THG can be modeled as follows^29,31,47^. Although wavelength tripling involves only virtual excited states, its efficiency is increased if real transitions (i.e. absorption bands) are present at frequencies *ω*, 2*ω* or 3*ω*. In this case, the third-order susceptibility can be expressed as:

3 $$\chi ^{(3)} = \chi _{nonresonant}^{(3)} + \chi _{resonant}^{(3)}$$

This formalism is similar to the one used in CARS microscopy^53^. Using the density matrix formalism and perturbation theory, the resonant term can be described as:

4 $$\chi _{res}^{\left( 3 \right)}\left( {3\omega {{{\mathrm{;}}}}\omega ,\omega ,\omega } \right) = \frac{N}{{\hbar}^{3}}\frac{{{\it{\mu }}_{ad}{\it{\mu }}_{dc}{\it{\mu }}_{cb}{\it{\mu }}_{ba}}}{{\left[ {\left( {\omega _{da} - 3\omega } \right) - i\gamma _{da}} \right]\left[ {\left( {\omega _{ca} - 2\omega } \right) - i\gamma _{ca}} \right]\left[ {\left( {\omega _{ba} - \omega } \right) - i\gamma _{ba}} \right]}}$$

where *N* is the atomic number density, μ_*mn*_ is the electric dipole moment between states *m* and *n* and *γ*_*mn*_ is the dephasing rate of the coherence between states *m* and *n*. The quantities ħ*ω*_*da*_, ħ*ω*_*ca*_, and ħ*ω*_*ba*_ correspond to three-, two- and one-photon resonances in the THG process associated with real transitions. The THG intensity associated to the resonant term $\chi _{res}^{\left( 3 \right)}$ is then given by:

5 $$I_{3\omega } \propto \left| {P^3\left( {3\omega } \right)} \right|^2 \propto \left| {\chi _{res}^{\left( 3 \right)}\left( {3\omega } \right)} \right|^2 \propto \frac{1}{{\left[ {\left( {\omega _{da} - 3\omega } \right)^2 + \gamma _{da}^2} \right]\left[ {\left( {\omega _{ca} - 2\omega } \right)^2 + \gamma _{ca}^2} \right]\left[ {\left( {\omega _{ba} - \omega } \right)^2 + \gamma _{ba}^2} \right]}}$$

It can be shown^40^ that the denominator terms are inversely proportional to the absorbance at the third-harmonic, second-harmonic and fundamental wavelengths. In turn, THG intensity can be altered by the presence of absorption at any of these frequencies. Chang et al.^31^ reported THG enhancement in oxyhemoglobin solutions in the 1200–1280 nm range, which can be explained by a dominant effect of absorption at the third-harmonic frequency, as the THG excitation spectrum correlates well with the linear absorption at 3*ω* in the Soret band spectral region. However, In the case of deoxyhemoglobin, the same study estimated a more complex spectrum suggesting a combined effect of 3P and 2P resonances.

### THG spectroscopy of RBCs

**Fig. 2:**
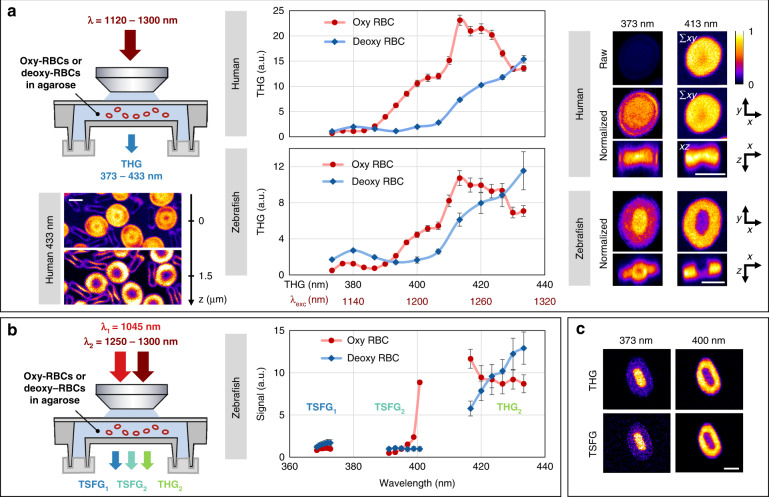
THG and TSFG spectral imaging of red blood cells near the Soret band. Average THG and TSFG signal from human and zebrafish RBCs in oxygenated and deoxygenated states. 3D images of individual RBCs were recorded at successive excitation wavelengths in the 1120–1300 nm range and normalized by the excitation power and pulse duration. A 10–20× signal enhancement is observed when the emission wavelength matches the Soret band, attributed to three-photon resonance. **a** Single-beam THG spectra and representative images at 433 nm (left, single planes) and at 373 and 413 nm (right, projections). The spectra were obtained from measurements on *N* = 7 human and *N* = 14 zebrafish RBCs. The images are normalized to their maxima except in the first line to illustrate the signal enhancement. The images shown are projections of the central planes along z and y directions. Unlike human RBCs, zebrafish RBCs possess a nucleus where no resonance is observed. **b** Dual-beam THG-TSFG spectra of zebrafish RBCs acquired by tuning the OPO beam in the range 1250–1300 nm. **c** Contrast similarity of single-beam THG (top) and TSFG (bottom) images. TSFG images at 373 and 401 nm were acquired simultaneously. Scale bars: 5 μm

Motivated by this rationale for the presence of THG resonances in hemoglobin, we performed a micro-spectroscopic characterization of THG from human or zebrafish isolated RBCs prepared in fully oxygenated and fully deoxygenated states in sealed chambers (see Methods). 3D THG images of individual RBCs were recorded at different wavelengths by tuning the OPO beam sequentially from 1120 to 1300 nm (Fig. 2a and S4). In the case of oxygenated RBCs, we used steps of 10 nm for the excitation wavelength (i.e. steps of ≈3.3 nm for THG wavelengths ranging from from 373 to 433 nm). In the case of deoxygenated RBCs, we increased the spectral step by a factor 2 in order to reduce the total imaging time to ≈20 min because RBCs rapidly capture oxygen under atmospheric conditions. Indeed, we observed with linear spectroscopy that after this delay, RBCs become partly oxygenated. Figure 2a presents average THG spectra extracted from the spectroscopic images from human and zebrafish RBCs in oxygenated and deoxygenated states, as well as typical images obtained at different wavelengths (see also Movie M1). One first remarkable observation is that THG from RBCs exhibits a very strong enhancement for excitation wavelengths in the range 1230–1300 nm, i.e. corresponding to the triple of the Soret band. THG from oxygenated RBCs is enhanced by a factor of ≈10–20 when the excitation is tuned from 1120 to 1240 nm, which is a considerable source of wavelength-dependent THG contrast. This efficient 3P resonance is consistent with THG measurements performed at a glass-hemoglobin interface^31^. As a consequence, THG contrast from individual RBCs is also modulated by this resonance: not only do RBCs look brighter at resonance, but the enhancement is specifically present around hemoglobin-rich areas. Indeed, unlike human RBCs, fish RBCs possess nuclei, which do not exhibit enhanced THG near 1240 nm (Fig. 2a, bottom right). Another important finding is that in deoxygenated RBCs, the THG enhancement peak is shifted towards larger wavelengths (≥1300 nm), most likely due to the shift of the Soret peak in deoxyhemoglobin. This data provides clear evidence of 3P resonant enhancement in THG signals from RBCs due to hemoglobin absorption in the Soret band, and of the sensitivity of these signals to the oxygenation state of hemoglobin.

### TSFG spectroscopy of RBCs

We then explored whether TSFG spectra exhibit the same wavelength dependence as THG, at the wavelengths accessible with our OPO system. We mounted oxygenated and deoxygenated RBCs in sealed chambers, and we recorded simultaneous TSFG_1_, TSFG_2_ and THG_2_ images while scanning the OPO wavelength from 1250 nm to 1300 nm with 10 nm increments. At each wavelength, we adjusted the delay line to ensure optimal matching of the pump and OPO pulses. Figure 2b shows the color TSFG spectra of oxy- and deoxygenated RBCs obtained for the three signals using this excitation mode. Their wavelength dependence is overall similar to corresponding portions of the spectra measured in single-beam THG mode (Fig. 2a) and are correlated with the linear absorption spectra of oxy- and deoxyhemoglobin (Fig. 1c). As illustrated in Fig. 2c and Fig. S5, individual images recorded on- and off-resonance were similar using single-beam THG and dual-beam TSFG excitation, with a resonant enhancement at the RBC periphery. These observations indicate that for RBCs imaging in the 1030–1300 nm range with 100–200 fs pulses, TSFG and THG signals exhibit similar phase-matching and resonance mechanisms. We also note that in the wavelength range accessible to our laser, the difference between signals from oxygenated and deoxygenated RBCs is maximized when the OPO wavelength is set to *λ*_2_ = 1300 nm, corresponding to the simultaneous detection of signals at 373, 401, and 433 nm. Figure 1c and S6 show the corresponding TSFG spectra and their position with respect to hemoglobin absorption spectra, given the pulse spectral bandwidth of our sources.

### RBC detection in zebrafish embryos

**Fig. 3:**
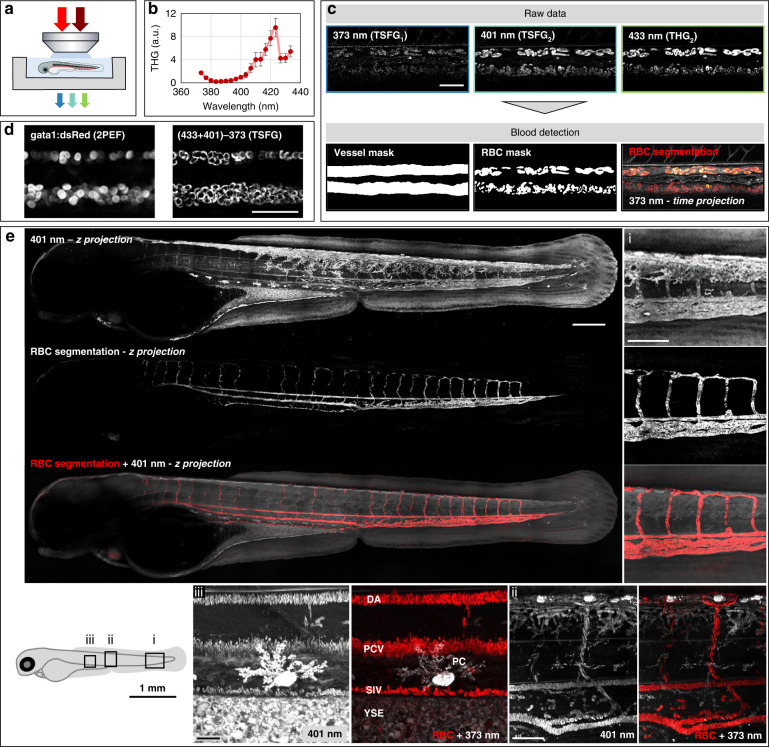
RBC detection in live zebrafish embryos with color TSFG microscopy. **a** Experimental scheme. **b** Average single-beam THG spectrum measured from flowing RBCs in live embryos (*N* = 3 embryos). **c** Segmentation of vessels and RBCs from color TSFG images acquired simultaneously at 373, 401 and 433 nm in a region encompassing DA and PCV in 2 dpf embryos. Scale bar: 50 μm. **d** Simultaneously acquired TSFG and fluorescence images of RBCs in a 2 dpf Tg(gata1a:DsRed) embryo. In this experiment, we induced transient cardioplegia using MS222 to immobilize the RBCs. Scale bar: 50 μm. **e** TSFG image of an entire embryo recorded in a tile-like fashion over a thickness of 100 µm. Shown are a z-projection of the 401 nm channel revealing blood vessels and other structures, the automated segmentation of the vascular system (see text and methods), and the overlay with the blood shown in red. (i), (ii) and (iii) show zoomed-in details of the tail and trunk, illustrating the effective discrimination provided by color TSFG signals between red blood cells and other structures producing strong signals such as pigmented cells, lipidic structures and other interfaces. DA dorsal aorta, PCV posterior cardinal vein, SIV subintestinal vein, PC pigment cell, YSE yolk sac extension. See also Movies M3–6, and supplementary Figs. S7–S8

We now discuss the identification of RBCs in TSFG images. One specific advantage of our scheme is that three signals are acquired simultaneously, so that spectroscopic analyses can be performed on images of moving objects. We analyzed how this can be used for automated segmentation of flowing RBCs in live zebrafish embryos. Two days post fertilization (dpf) *casper* embryos^54^ were mounted on their lateral side (see Methods) to image the dorsal aorta (DA) and the posterior cardinal vein (PCV) (Fig. 3a).

The observed THG signal level for individual RBCs using OPO excitation in our conditions (30–40 mW; NA = 1; 80 MHz; 140 fs) was on the order of 2 × 10^6^ photons/s, i.e. compatible with dynamic imaging with ~ 5 µs pixel dwell times. We first recorded time series of single-beam THG images at different wavelengths to confirm that the THG spectrum of flowing RBCs is consistent with that of isolated RBCs (Fig. 3b). Since that spectrum exhibited a relative minimum at 430 nm, we concluded that RBCs at this stage of the embryo development are in a largely oxygenated state, consistent with the literature^55^.

We then acquired time series of simultaneous TSFG-THG images. A typical three-channel image extracted from a time series is shown in Fig. 3c. The vessel closer to the notochord (upper part in the images) is the artery (dorsal aorta, DA). Due to the pulsed arterial flow and constant laser scanning speed, some RBCs in the artery appear elongated in the images. In contrast, blood flow in the vein is constant. The TSFG_1_ signal level at 373 nm is overall weaker than the two other channels due to reduced optics transmission and absence of hemoglobin resonance in this wavelength range. Nevertheless, flowing RBCs can be imaged simultaneously in the three channels despite their motion during image acquisition, along with other structural features. When using mixed 1045 and 1300 nm excitation, the TSFG_2_ and THG_2_ signals detected at 401 (*S*_401_) and 433 nm (*S*_433_) lie in the resonant part of the hemoglobin spectrum. In contrast, the TSFG_1_ signal detected at 373 nm (*S*_373_) is away from the Soret resonance (Fig. 1c). An “Enhancement” image can be generated using simple combinations of the three signals to isolate RBC-specific contributions (see Methods). We could then segment blood vessels and RBCs (Fig. 3c and Movie M2) by generating binary masks from “Enhancement” images following straightforward image processing steps (see Methods). Remarkably, RBC pixels could be automatically detected at each time point by taking advantage of the simultaneity of the spectroscopic imaging process. We confirmed the specificity of RBC detection by comparing color TSFG and fluorescence of *Tg(gata1a:DsRed)* zebrafish embryos exhibiting red fluorescence in RBCs^56^. The 2PEF signal was excited by the 1045 nm beam and simultaneously collected on a fourth channel in epi-detection. The similarity between the two images (Fig. 3d) confirms the specificity of TSFG imaging for RBC detection based on resonant TSFG in hemoglobin. Of note, after manually counting 576 cells, we found that 48 cells (8.3 %) were visible in TSFG but did not exhibit fluorescence. This is consistent with the reported properties of the *Tg(gata1a:DsRed)* line^56^ and illustrates one potential advantage of label-free imaging, which is that it does not miss unlabeled cells.

To further illustrate the potential of TSFG microscopy for blood detection, we imaged an entire 3 dpf wild-type zebrafish embryo (Fig. 3e and Movie M3), in which melanin pigmentation was chemically prevented^57^. A mosaic of 33 3D tiles (418 μm × 418 μm × 100 μm) was recorded and stitched to reconstruct a large volume (1.13 mm × 3.97 mm × 0.10 mm). We then segmented RBCs as described above (see also Methods). This dataset illustrates that the vascular system can be detected in the entire embryo with high specificity (Fig. 3e zoom (i) or Fig. S7, Movie M4), even in the presence of other sources of THG contrast such as myelinated axons (Movie M5), pigmented cells (Fig. 3e zoom (ii), Fig. S8 and Movie M6), or lipid bodies from the yolk (Fig. 3e, zoom (iii) or Fig S8b). Overall, these results establish a novel label-free technique allowing automated segmentation of flowing RBCs and blood vessels in zebrafish embryos.

### Probing oxygenation with color TSFG

**Fig. 4:**
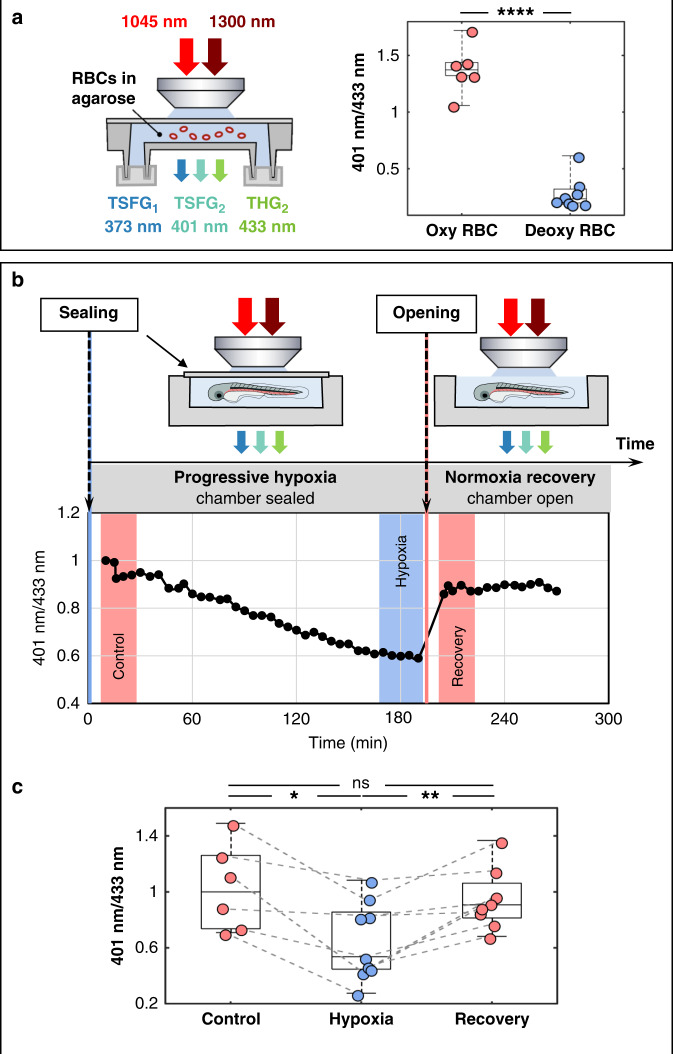
Probing RBC oxygenation state with color TSFG microscopy. **a** Experimental setup and measured S_401_/S_433_ TSFG signal ratios for isolated zebrafish RBCs in oxygenated (*N* = 6) and deoxygenated (*N* = 8) states. *p* ≤ 0.0001 (unpaired two-tailed Whelch’s test). **b** Oxygenation dynamics measured on flowing RBCs in a 2 dpf zebrafish. The S_401_/S_433_ ratio was measured every 5 min during progressive hypoxia in a sealed chamber and after chamber opening. **c** Evolution of the S_401_/S_433_ ratio during reversible hypoxia experiments for *N* = 9 different embryos. The values are the average of several points spanning 25 min as in the colored regions shown in the graph in **b**. The values were normalized by setting the median of the control to 1. One star: *p* ≤ 0.05; two stars: *p* ≤ 0.01 (mixed-effects model)

Elaborating on the ability to detect RBCs with high specificity based on their color TSFG response, we checked whether it is also possible to probe their oxygenation state using this signal. We first analyzed immobilized RBCs in fully oxygenated and fully deoxygenated states. Based on the spectra in Fig. 2, we proposed to use the ratio of the signals measured at 401 nm and 433 nm as a simple reporter of oxygenation, and defined an oxygenation parameter as $R = S_{401}/S_{433} = TSFG_1/THG_2$. Thus, *R* is expected to be highest for RBCs in an oxygenated state. We estimated *R* from each segmented RBC image by plotting the signal from pixels at 401 and 433 nm and performing a linear regression. As anticipated, oxygenated RBCs exhibit a higher value of the *R* parameter than deoxygenated ones (Fig. 4a), which demonstrates that the oxygenation state of isolated RBCs can be probed with color TSFG microscopy.

We extended this approach to in vivo imaging by analyzing TSFG signals in 2 dpf zebrafish embryos submitted to artificial hypoxia. Embryos were mounted as described previously except that the observation chamber was sealed (Fig. 4b). The embryo and agarose were deposited in a ≈ 100 µL well at the bottom of the microscope slide, and covered with a coverslip sealed with silicone (twinsil speed, Picodent, Germany) while the agarose was still liquid. Within 10 min after mounting, the imaging session started and consisted in recording a time series of 30 images every 5 min during several^2–5^ hours. After that session, the top cover glass of the chamber was removed to recover normoxia, the dish was filled with embryo medium and placed back under the microscope with the embryo still embedded in agarose. The same region was then imaged. The time between the last hypoxia image and the first one after opening chamber was <15 min. From the recorded images, we segmented the RBCs and extracted the average oxygenation parameter *R* = *S*_401_/*S*_433_ at each time point. Figure 4b shows the time evolution of *R* in the case of one embryo. The values of *R* before, during, and after hypoxia for nine embryos is presented in Fig. 4c. We consistently observed a decrease of the *R* parameter during the hypoxia assay (typically—40% after 3 h), suggesting that the limited oxygen supply inside the small chamber volume was being consumed by the embryo. After chamber opening, the *R* parameter returned to its original value, indicating that RBCs recovered their oxygenation state within minutes after hypoxia. This fast recovery is consistent with^58^. Together, these data provide strong evidence for the possibility of label-free functional TSFG microscopy. Importantly, our implementation can probe RBCs in vivo with single-cell resolution and microsecond pixel dwell times.

### Deep-tissue blood imaging in adult zebrafish brain

**Fig. 5:**
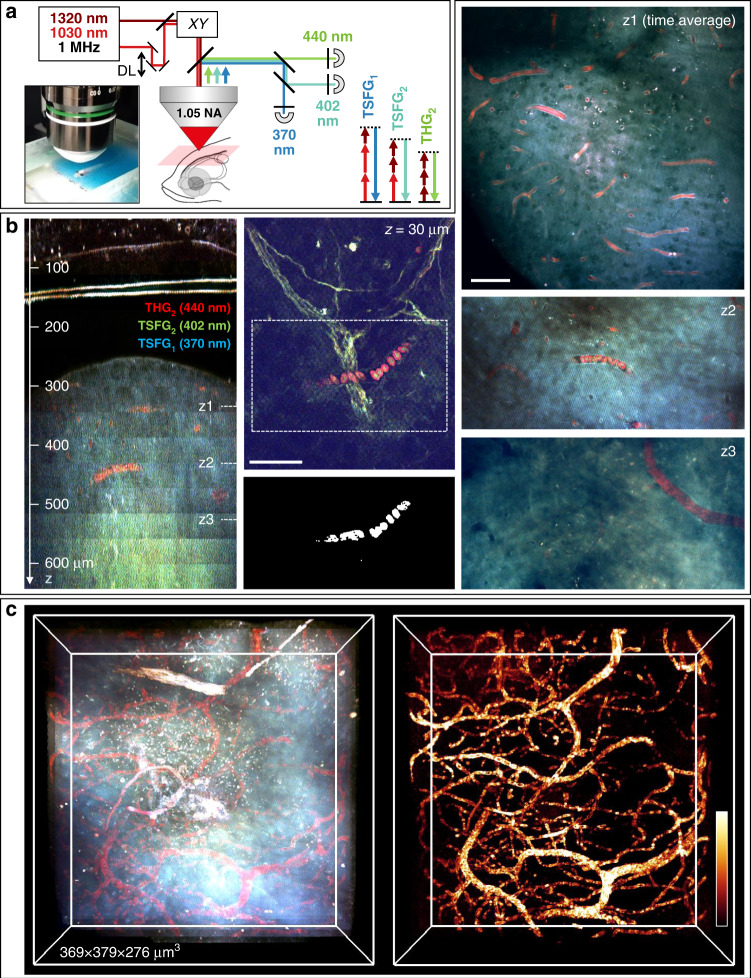
Deep-tissue TSFG imaging in live adult zebrafish brain. **a** TSFG microscopy setup scheme for deep tissue imaging with 1 MHz excitation and signal epi-detection. **b** (left) XZ reslice calculated from a color TSFG z-series spanning >600 μm of imaging depth through the skin, skull and telencephalon of an adult fish. (middle) Sub-skin image showing different spectral signatures for RBCs (in red) and myelinated axons. Segmented RBCs are shown below the image. (Right) Representative images recorded at several depths in the telencephalon, illustrating the specific TSFG contrast of RBCs and vessels. **c** 3D rendering of the imaged volume in the telencephalon and of the segmented blood vessels. Scale bar: 50 μm. See also Movies M7–10

Finally, we transposed TSFG microscopy to deep-tissue imaging by implementing this contrast modality on an OPA-based microscope and adapting it to epi-detection (Fig. 5a). The pump and OPA wavelengths were centered at 1030 and 1320 nm respectively, resulting in TSFG and THG wavelengths at $\lambda _{TSFG_1} = 370\,nm$, $\lambda _{TSFG_2} = 402\,nm$ and $\lambda _{THG_2} = 440\,nm$. We remind that OPA-based excitation at 1 MHz is expected to provide an 80^2^ increase in THG efficiency compared to 80 MHz OPO excitation with similar pulse durations and average power, enabling to work at larger depths by progressively increasing the power delivered to the tissue surface^59^. One difference with the transmission geometry used in the previous section is that the epidetected signals correspond to THG and TSFG light that is mostly forward-generated and then scattered back towards the objective by tissue structures underneath the imaging plane. Contrast can only be present to the extent that the signal photons are not significantly reabsorbed on their way to the tissue surface^20,60^. An adult *casper* zebrafish was anesthetized and held between two pieces of foam under the microscope objective positioned above the telencephalon (Fig. 5a). To confirm that we could detect hemoglobin-specific TSFG enhancement in this geometry, we first imaged superficial blood vessels located above the skull, which is located ~150 µm under the skin surface. An example is shown in Fig. 5b (middle) and Movie M7. As in the embryo experiments with OPO excitation, the signal at 440 nm is enhanced in RBCs compared to the signal at 370 nm. We note, however, that the resonance at 402 nm is less pronounced than in the OPO experiments, which may be due to the broader spectral bandwidth of the OPA pulses and/or slightly different wavelengths involved of the two systems. Nevertheless, overlaying the three channels clearly highlights RBCs from the other structures visible in THG-TSFG images such as myelinated fibers. We verified that we could automatically segment RBCs in such images by using an “Enhancement” image calculation (Fig. 5b middle).

We then evaluated the potential of TSFG to detect RBCs at larger depths. We recorded a *z*-series spanning ≈650 µm of imaging depth which encompassed the skin, the skull (≈150–180 µm under the surface), the choroid plexus, cerebrospinal fluid, and a fraction of the telencephalic parenchyma (250–650 µm under the surface), while progressively increasing the excitation power *P*. For visualizing the *z*-series, we fitted each channel *z*-profile with an exponential function of the form $S = S_0 \ast P^3{{{\mathrm{exp}}}}( - 3z/EAL)$ and re-normalized the images by this function. The *EAL* parameter is usually termed the effective attenuation length and can be interpreted as an estimate of the scattering length of excitation light in the tissue^61,62^. In the telencephalon, we measured *EAL* to be 165 ± 10 µm for 1320 nm excitation and 135 ± 10 µm for mixed 1030/1320 nm excitation. These numbers are overall consistent with values reported in other organisms at 1300 nm, such as 200 µm in Danionella brain^28^ and 300 µm in mouse cortex^63^. Our data confirms that a specific TSFG contrast is observed from RBCs and blood vessels at least until depths of 600 µm (Fig. 5b and Movie M8). Figure 5c and Movie M9 present volume renderings of the three independent signals and a composite. As in the case of zebrafish embryos, a single channel (*e.g.* THG at 440 nm) is not sufficient to unambiguously identify RBCs (Fig. 5c left). The selective segmentation of RBCs and blood vessels is possible only due to the multichannel nature of our TSFG imaging approach. Automated RBC segmentation was possible over the entire imaged fraction of the telencephalon, highlighting the vascular network (Fig. 5c right and Movies M9–10). We note that other structures such as myelinated fibers were also detected until depths exceeding 600 µm, and exhibited ratios between TSFG channels different from RBCs.

In this experiment, the maximum average powers delivered after the objective when imaging the deepest planes were *P*_pump_ = 61 mW and *P*_opa_ = 74 mW. This total power remains compatible with previous recommendations for in vivo imaging^62^. Adult fish (*N* = 3) recovered from anesthesia and exhibited normal swimming behavior after 90 min of imaging experiments. In addition, we point out that relatively long pulses (180–200 fs) were used in this experiment, and that similar THG/TSFG signal levels would be obtained with half this average power if using pulse durations in the 60 fs range.

Overall, these experiments demonstrate that our color TSFG scheme can be implemented on an OPA-based microscope such as those required for 3-photon microscopy, and is compatible with epi-detection. This implementation provides label-free hemoglobin specific imaging of blood vessels and RBCs deep inside a live adult zebrafish brain.

## Discussion

In this work, we have introduced and demonstrated a novel experimental scheme for color TSFG imaging with simultaneous detection at three wavelengths. We have also established that there is a strong 3P resonance due to the Soret band of hemoglobin in THG and TSFG signals from fish and human RBCs with excitation in the 1250–1300 nm range. This resonance causes a 10–20× increase in signal at resonance, and is spectrally shifted depending on RBC oxygenation state. This is an important ground observation that complements previous studies, and brings important perspectives for multiphoton imaging based on *χ*^(3)^ contrast. We have shown that our color TSFG scheme enables specific detection of RBCs, and that it can be efficiently implemented on a 3P microscope. From this perspective, it extends the contrast possibilities of 3P microscopy by adding a label-free hemoglobin-specific contrast, and it extends the contrast possibilities of THG microscopy by providing a way to separate blood from other visible structures such as myelin. Importantly, this contrast modality appears to be sensitive to RBC oxygenation, and possesses some very distinctive characteristics compared to other existing methods: our data shows that TSFG (i) provides single-cell resolution even in the context of deep-tissue microscopy; (ii) requires only microsecond pixel durations; (iii) is a label-free method; (iv) directly probes RBCs rather than blood plasma; (v) can probe moving objects such as flowing RBCs thanks to the simultaneous ratiometric measurement at several wavelengths. TSFG signal levels with OPA excitation are comparable to 3P fluorescence signals and are therefore compatible with rapid in vivo imaging.

These findings should motivate additional developments and applications. First, it will be interesting to investigate which wavelengths and pulse bandwidths optimize the sensitivity of color TSFG to RBCs and their oxygenation state, in particular when using OPA excitation. For deep-tissue applications, it will be relevant to analyze the possibility of THG/TSFG partial reabsorption by the tissue during backscattering towards the objective, and to evaluate to what extent this phenomenon can alter the spectral measurements. It will also be interesting to explore how complementary the information obtained with TSFG, where RBC are directly probed, is complementary to that obtained with other approaches such as plasma-injected phosphorescent reporters.

A particularly appealing perspective of our work is to combine TSFG with three-photon fluorescence imaging. For example, combining TSFG with calcium imaging using a fluorescent reporter should result in a novel method to investigate neurovascular interactions at the circuit scale. This technical development is relatively straightforward, since efficient 3P excitation of GCaMP reporters is obtained with 1300–1320 nm excitation^64^ already used in our TSFG scheme. An additional perspective is to use color TSFG to achieve label-free selective detection of other non-fluorescent absorbers and pigments in tissues. Although the implementation of TSFG requires to overlap and synchronize two pulsed beams, the technical challenge is not greater than in the cases of CARS and wavelength-mixing two-photon microscopies, which both have been successfully used for life science studies^53,65^. We also remind that the second beam is simply derived from the pump laser already available on THG and 3 P microscopes. One potential limitation of TSFG obviously comes from the extra-illumination associated with the second beam, which may shift the speed/depth compromise^13,62^ unfavorably compared to single-beam methods. However, dual-beam excitation comes with the benefit of giving simultaneous access to several additional nonlinear signals^35,37,66^ and biological parameters.

Overall, we have demonstrated a novel contrast modality of label-free multiphoton microscopy, along with a novel implementation which is directly compatible with deep-tissue 3P microscopy. This work should open the way to a variety of applications in neuroscience and physiology.

## Materials and methods

### Microscopy

Experiments were performed on a lab-built upright multiphoton microscope equipped with galvanometric scanners (GSI Lumonics, USA), a water immersion objective (25×, 1.05 NA, Olympus, Japan), and a dual output femtosecond laser. Pulses from the two laser beams were coaligned, overlapped spatially in the axial direction using two independent telescopes, and synchronized temporally using a motorized delay line (ODL220/M, ThorLabs, USA). Two different laser systems were used in this work, termed ‘OPO’ and ‘OPA’. For experiments on isolated RBCs and on zebrafish embryos, excitation was provided by a dual output femtosecond 80 MHz laser source (Insight X3, Spectra Physics, USA) providing a fixed output at 1045 nm (‘pump’) and a wavelength-tunable output at 1100–1300 nm (‘OPO’). This laser system has the advantage of continuous wavelength tunability, which was useful to characterize the THG-TSFG micro-spectroscopy of RBCs and to determine optimal wavelengths for color imaging. Pulse duration at the sample plane was measured to be 240 fs for the pump and 110 to 160 fs for the OPO (Fig. S2b). Pulse spectral bandwidth was ~5.5 nm for the pump and 7 nm for the OPO. THG and TSFG signal collection was performed in transmission using a high NA condenser (Olympus, Japan), dichroic mirrors (FF414-Di01 and FF389-Di01, Semrock, USA), filters (FF01-373/10, FF01-400/12, FF01-434/17, Semrock, USA) and three photomultiplier modules (P25PC, Sens-Tech, UK). Detection was performed using lab-designed MHz-rate counting electronics. The signal level was kept in a range avoiding photon piling, i.e. less than 1 detected photon every 4 laser pulses. For experiments on adult zebrafish, excitation was provided by a 1 MHz femtosecond OPA prototype (Satsuma NIJI, Amplitude, France) providing two output beams at 1030 nm (‘pump’) and 1320 nm (‘OPA’). This laser system has limited wavelength tunability, but has the advantage of providing higher peak power resulting in larger THG-TSFG signals compared to the OPO system for the same average power and pulse duration, in turn enabling deep-tissue imaging. Pulse duration at the sample was measured to be 250 fs for the pump and 180 fs for the OPA. Pulse spectral bandwidth was ~6 nm for the pump and 19 nm for the OPA. THG and TSFG signals were collected in epi-detection through the excitation objective, separated from the excitation using a dichroic (longpass filter at 850 nm, Chroma Technology, USA), and directed on three GaAsP photodetectors (H7422P-40, Hamamatsu, Japan) using the dichroics and filters described previously. Detection was performed in analog mode using lab-designed electronics. The acquisition was controlled using LabView-written software (National Instruments, USA) except for mosaic images of zebrafish embryos (Fig. 3e and Movies M3–6) and for adult zebrafish experiments (Fig. 5 and Movies M7–10), for which we used ScanImage acquisition software (Vidrio inc, USA). Images were acquired at a pixel dwell time of 5 and 6 µs for LabView and ScanImage, respectively. For specific acquisition conditions, see Supplementary table T1.

### RBCs preparation and imaging

Human RBCs were obtained from full blood samples of different anonymized patients (Cerba Xpert, France). Zebrafish RBCs were extracted from wild-type fish from our facility following the procedure in^67^. RBCs are fully oxygenated under atmospheric conditions. To prepare them for imaging, ~20 μL of blood were diluted in 200 μL of an isotonic solution. The result was used to prepare a 5 mg/mL agarose gel to prevent the cells from moving during imaging and mounted in a channel slide (IBIDI, Germany). Low melting agarose was used to keep the cells at physiological temperature. To obtain RBCs in deoxygenated state, the isotonic solution containing RBCs in agarose was degassed and put under argon while kept at 42 °C. A solution of a reducing agent (Na2S2O4, 92 mM in isotonic solution) was prepared and also degassed in vacuum (50 Pa) and kept under argon (1.3 × 10^5^ Pa). It was injected in the agarose solution containing RBCs resulting in a final concentration of 4.4 mM and 19 mg/mL in agarose. The sealed channel was also degassed in vacuum and filled with argon. The deoxygenated RBCs in solution were transferred with a gastight syringe inside the channel through the rubber cap, which was finally sealed with vacuum silicone paste. This provided sufficient tightness to keep the cells in deoxygenated state for the duration of the imaging session (20 min). Linear absorption spectra were measured on a spectrophotometer before and after THG-TSFG imaging to confirm the RBCs’ oxygenation state and its stability (Fig. 1c).

Isotonic solution for human RBCs: [NaCl] = 140 mM; [KCl] = 2.7 mM; [CaCl_2_] = 1.2 mM; [glucose] = 5.5 mM. pH = 7.8.

Isotonic solution for zebrafish RBCs: [NaCl] = 145 mM; [KCl] = 5 mM; [CaCl_2_] = 1.4 mM; [MgSO_4_] = 1 mM; [Hepes] = 15 mM; [D-Glucose] = 5 mM; pH = 7.9.

At each wavelength, we recorded z-stacks with 1 µm voxel depth, and 20–45 mW of excitation power. To avoid artefacts due to THG-TSFG light reabsorption by hemoglobin, we imaged cells located near the bottom of the chamber, (i.e. with no cells underneath). Images were normalized according to excitation power and pulse duration dependences given by Eq. 1. For example, THG images were divided by the cube of the laser power and multiplied by the square of the pulse duration for each wavelength.

### Zebrafish embryo imaging

Zebrafish embryos were obtained by natural spawning of AB, *Tg(gata1a:DsRed)* and *casper* (depigmented) fish^54^. Before the imaging session, the embryos were anesthetized with 0.16 mg/mL MS222 (Sigma-Aldrich, USA) in embryo medium, dechorionated and mounted in a low melting point agarose gel at 5 mg/mL in 0.16 mg/mL MS222. The embryo and the gel added up to a ≈ 100 μL volume fitting the bottom well of a glass bottom dish allowing transmission imaging. Embryos were mounted with their anteroposterior axis orthogonal to the fast scan axis. In this orientation, the blood flow was also perpendicular to the fast scan axis, which allowed imaging more RBCs. For experiments in normoxic conditions, the dish was filled with anesthetic embryo medium^68^, 0.16 mg/mL in MS222, used as the immersion medium of the objective. For experiments in progressive hypoxic conditions, the well was covered with a cover glass sealed with silicone (twinsil speed, Picodent) while the agarose was still liquid. The total amount of power used on the embryos did not exceed 60 mW.

### Adult zebrafish imaging

Live adult zebrafish imaging was performed using the protocol described in ref. ^66^. Twelve-months-old casper (depigmented) zebrafish^54^ were used in the study. Anesthesia was initiated by soaking the fish for 90 s in water containing 0.2 mg/mL MS222 (Sigma-Aldrich, USA). Fish were then transferred into a water solution of 0.1 mg/mL MS222 and 0.05 mg/mL isoflurane to maintain the anesthesia during imaging, mounted in a plastic dish and held between pieces of sponge. Signals were epidetected using the excitation objective. The acquisition consisted of 12 successive z-stacks for which the excitation power was progressively increased with depth, and the z-step between successive images was 2 µm. We normalized the successive z-stacks by the excitation powers according to the dependencies of TSFG signals discussed in Eq. 1, and z-stitched the processed stacks. Overall, one imaging session lasted up to 90 min. We used a total excitation power up to 135 mW at the sample surface at the largest imaging depths. All animal experiments were conformed to French and European ethical and animal welfare directives (project authorization from the Ministère de l’Enseignement Supérieur, de la Recherche et de l’Innovation to N. Dr.).

### Polystyrene beads in agarose gels

To calibrate the power dependence of THG and TSFG signals, we used 0.5 µm non-fluorescent polystyrene beads (Sigma-Aldrich, USA). To calibrate lateral chromatic aberrations and resolution, we used 0.5 µm fluorescent beads (TetraSpeck Microspheres, ThermoFisher Scientific, USA). Beads were included in a 20 mg/mL low melting point agarose solution. While still liquid, a small volume of the solution was mounted between a glass and a cover glass. The edges of the cover glass were sealed with silicone (twinsil speed, Picodent, Germany). We imaged beads located away from the top and the bottom of the gel in order to avoid the THG signal from agarose-glass interfaces.

### Image processing

To process and analyze the images in this work we used ImageJ, MATLAB (MathWorks, CA, USA) and Imaris (Bitplane, Switzerland). For RBC segmentation, several sequences of image operations were tested and successfully used to generate “Enhancement” maps. To segment flowing RBCs in 2 dpf embryos around the ROI shown in Fig. 3c and in hypoxia experiments (Fig. 4b), we used Enhancement = (*S*_401_ + *S*_433_)/*S*_373_. This definition allows the detection of RBC pixels through the normalization of an image containing resonant hemoglobin THG-TSFG signals by a non-resonant one. In experiments where the non-resonant signal at 373 nm was weak (as in Fig. 3d), we used the alternative definition Enhancement = (*S*_401_ + *S*_433_) − *S*_373_, in order to minimize noise propagation. For processing the large-scale mosaic image (Fig. 3e), we used the simplified option Enhancement = *S*_401_ − *S*_373_. Finally, in the case of adult zebrafish brain images with OPA excitation, we found that the signal at 402 nm exhibited only limited resonance and we used Enhancement = *S*_440_/(*S*_402_ + *S*_370_) or Enhancement = *S*_440_ − *S*_402_. To compute the “vessel” binary mask in Fig. 3c we applied a binary threshold to the time maximum projection of the image series. The “RBC” binary mask in Fig. 3c and Movie M2 was then obtained at each time point by first multiplying the “Enhancement” time series by the “vessel” binary mask, then applying a Gaussian filter and a binary threshold, and finally a dilation-erosion binary filter to remove isolated pixels.

### Statistical analysis

Statistical analyses were done using Prism (GraphPad software, CA, USA). We used an unpaired two-tailed Welch’s test for the data in Fig. 4a. Because the experiments on different embryos were parallelized, only specific time points were acquired for several fish in Fig. 4c instead of imaging through the whole dynamics of the process as in Fig. 4b. For this reason, in Fig. 4c we used a mixed-effects model to test the data.

### INSTITUTIONAL REVIEW BOARD (IRB): IRB institution name and number

Comité d’éthique en expérimentation animale Paris Centre et Sud—CEEA N°59. Project authorizations numbers delivered by the Ministère de l’Enseignement Supérieur, de la Recherche et de l’innovation: CODECOH DC_2018_3300; 23641.

## Supplementary information

Supplementary Information

Movie M1. THG spectral imaging of RBCs

Movie M2. RBC detection in 2 dpf zebrafish embryos

Movie M3. 3D map of the vascular system in the entire 3 dpf wild type embryo

Movie M4. Specific detection of RBCs among TSFG signals from other structures in the trunk

Movie M5. Specific detection of RBCs among TSFG signals from surrounding structures and myelinated axons

Movie M6. Specific detection of RBCs among nearby pigmented cells

Movie M7. RBC dynamics in a live adult zebrafish

Movie M8. Label-free color TSFG images recorded at successive depths in a live adult zebrafish brain

Movie S9. 3D rendering of an adult zebrafish telencephalon

Movie M10. 3D rendering of the vasculature in an adult zebrafish telencephalon

## Data Availability

All data needed to evaluate the conclusions in the paper are present in the paper and/or the Supplementary Materials. Additional data related to this paper may be requested from the authors or the LOB data access committee at data.lob@meslistes.polytechnique.fr.

## References

[1] Kleinfeld D (1998). Fluctuations and stimulus-induced changes in blood flow observed in individual capillaries in layers 2 through 4 of rat neocortex. Proc. Natl Acad. Sci. USA.

[2] Chaigneau E (2003). Two-photon imaging of capillary blood flow in olfactory bulb glomeruli. Proc. Natl Acad. Sci. USA.

[3] Shih AY (2012). Two-photon microscopy as a tool to study blood flow and neurovascular coupling in the rodent brain. J. Cerebral Blood Flow Metabol..

[4] Finikova OS (2008). Oxygen microscopy by two-photon-excited phosphorescence. ChemPhysChem.

[5] Sakadžić S (2010). Two-photon high-resolution measurement of partial pressure of oxygen in cerebral vasculature and tissue. Nat. Methods.

[6] Lecoq J (2011). Simultaneous two-photon imaging of oxygen and blood flow in deep cerebral vessels. Nat. Med..

[7] Esipova TV (2019). *Oxyphor* 2P: a high-performance probe for deep-tissue longitudinal oxygen imaging. Cell Metabol..

[8] Zeng Y (2014). In vivo micro-vascular imaging and flow cytometry in zebrafish using two-photon excited endogenous fluorescence. Biomed. Optics Express.

[9] Theer P, Denk W (2006). On the fundamental imaging-depth limit in two-photon microscopy. J. Optical Soc. Am. A.

[10] Horton NG (2013). In vivo three-photon microscopy of subcortical structures within an intact mouse brain. Nat. Photon..

[11] Ouzounov DG (2017). In vivo three-photon imaging of activity of GCaMP6-labeled neurons deep in intact mouse brain. Nat. Methods.

[12] Guesmi K (2018). Dual-color deep-tissue three-photon microscopy with a multiband infrared laser. Light Sci. Appl..

[13] Yildirim M (2019). Functional imaging of visual cortical layers and subplate in awake mice with optimized three-photon microscopy. Nat. Commun..

[14] Streich L (2021). High-resolution structural and functional deep brain imaging using adaptive optics three-photon microscopy. Nat. Methods.

[15] Barad Y (1997). Nonlinear scanning laser microscopy by third harmonic generation. Appl. Phys. Lett..

[16] Müller M (1998). 3D microscopy of transparent objects using third-harmonic generation. J. Microscopy.

[17] Oron D (2004). Depth-resolved structural imaging by third-harmonic generation microscopy. J.Struct. Biol..

[18] Olivier N (2010). Cell lineage reconstruction of early zebrafish embryos using label-free nonlinear microscopy. Science.

[19] Farrar MJ (2011). In vivo imaging of myelin in the vertebrate central nervous system using third harmonic generation microscopy. Biophys. J..

[20] Débarre D (2006). Imaging lipid bodies in cells and tissues using third-harmonic generation microscopy. Nat. Methods.

[21] Weigelin B, Bakker GJ, Friedl P (2016). Third harmonic generation microscopy of cells and tissue organization. J. Cell Sci..

[22] Sun CK (2004). Higher harmonic generation microscopy for developmental biology. J. Struct. Biol..

[23] Tserevelakis GJ (2014). Label-free imaging of lipid depositions in *C. elegans* using third-harmonic generation microscopy. PLoS One.

[24] Lim H (2014). Label-free imaging of Schwann cell myelination by third harmonic generation microscopy. Proc. Natl Acad. Sci. USA.

[25] Witte S (2011). Label-free live brain imaging and targeted patching with third-harmonic generation microscopy. Proc. Natl Acad. Sci. USA.

[26] Liu HJ (2020). In vivo deep-brain blood flow speed measurement through third-harmonic generation imaging excited at the 1700-nm window. Biomed.Optics Express.

[27] Chow DM (2020). Deep three-photon imaging of the brain in intact adult zebrafish. Nat. Methods.

[28] Akbari, N. et al. Whole-brain optical access in small adult vertebrates with two- and three-photon microscopy. *bioRxiv* (in the press). 10.1016/j.isci.2022.105191 PMC9557827 36248737

[29] Clay GO (2006). Spectroscopy of third-harmonic generation: evidence for resonances in model compounds and ligated hemoglobin. J. Opt. Soc. Am. B.

[30] Yu CH (2008). Molecular third-harmonic-generation microscopy through resonance enhancement with absorbing dye. Opt. Lett..

[31] Chang CF, Yu CH, Sun CK (2010). Multi-photon resonance enhancement of third harmonic generation in human oxyhemoglobin and deoxyhemoglobin. J. Biophoton..

[32] Tripathy U (2013). Optimization of malaria detection based on third harmonic generation imaging of hemozoin. Anal. Bioanal. Chem..

[33] Segawa H (2014). Electronically resonant third-order sum frequency generation spectroscopy using a nanosecond white-light supercontinuum. Opt. Express.

[34] Lanin AA (2019). Three-photon-resonance-enhanced third-harmonic generation for label-free deep-brain imaging: In search of a chemical contrast. J. Raman Spectrosc..

[35] Campargue G (2020). Multiorder nonlinear mixing in metal oxide nanoparticles. Nano Lett..

[36] Hanninen AM (2018). High-resolution infrared imaging of biological samples with third-order sum-frequency generation microscopy. Biomed. Opt. Express.

[37] Mahou P (2012). Multicolor two-photon tissue imaging by wavelength mixing. Nat. Methods.

[38] Xu, C. & Webb, W. W. in *Topics in Fluorescence Spectroscopy* (ed Lakowicz, J. R.) 471–540 (Springer, 2002).

[39] Mahou P (2017). Metrology of multiphoton microscopes using second harmonic generation nanoprobes. Small.

[40] Boyd RW (2008). Nonlinear Optics..

[41] Cheng JX, Xie XS (2002). Green’s function formulation for third-harmonic generation microscopy. J. Opt. Soc. Am. B.

[42] Débarre D, Supatto W, Beaurepaire E (2005). Structure sensitivity in third-harmonic generation microscopy. Opt. Lett..

[43] Morizet J (2021). Modeling nonlinear microscopy near index-mismatched interfaces. Optica.

[44] Millard AC (1999). Third-harmonic generation microscopy by use of a compact, femtosecond fiber laser source. Appl. Opt..

[45] Barzda V (2005). Visualization of mitochondria in cardiomyocytes. Opt. Express.

[46] Tuer AE (2010). Nonlinear multicontrast microscopy of hematoxylin-and-eosin-stained histological sections. J. Biomed. Opt..

[47] Schaller RD, Johnson JC, Saykally RJ (2000). Nonlinear chemical imaging microscopy: near-field third harmonic generation imaging of human red blood cells. Anal. Chem..

[48] Rehberg M (2011). Label-free 3D visualization of cellular and tissue structures in intact muscle with second and third harmonic generation microscopy. PLoS One.

[49] Weigelin B, Bakker GJ, Friedl P (2012). Intravital third harmonic generation microscopy of collective melanoma cell invasion: principles of interface guidance and microvesicle dynamics. IntraVital.

[50] Dietzel S (2014). Label-free determination of hemodynamic parameters in the microcirculaton with third harmonic generation microscopy. PLoS One.

[51] Ahn SJ (2020). Label-free assessment of hemodynamics in individual cortical brain vessels using third harmonic generation microscopy. Biomed. Opt. Express.

[52] Chen CK, Liu TM (2012). Imaging morphodynamics of human blood cells in vivo with video-rate third harmonic generation microscopy. Biomed. Opt. Express.

[53] Zhang C, Zhang DL, Cheng JX (2015). Coherent Raman scattering microscopy in biology and medicine. Ann. Rev. Biomed. Eng..

[54] White RM (2008). Transparent adult zebrafish as a tool for in vivo transplantation analysis. Cell Stem Cell.

[55] Grillitsch S (2005). J. Exp. Biol..

[56] Traver D (2003). Transplantation and in vivo imaging of multilineage engraftment in zebrafish bloodless mutants. Nat. Immunol..

[57] Karlsson J, Von Hofsten J, Olsson PE (2001). Generating transparent zebrafish: a refined method to improve detection of gene expression during embryonic development. Mar. Biotechnol..

[58] Huang SH, Yu CH, Chien YL (2015). Light-addressable measurement of in vivo tissue oxygenation in an unanesthetized zebrafish embryo via phase-based phosphorescence lifetime detection. Sensors..

[59] Bakker GJ (2022). Intravital deep-tumor single-beam 3-photon, 4-photon, and harmonic microscopy. eLife.

[60] Débarre D, Olivier N, Beaurepaire E (2007). Signal epidetection in third-harmonic generation microscopy of turbid media. Opt Express.

[61] Wang MR (2018). Comparing the effective attenuation lengths for long wavelength in vivo imaging of the mouse brain. Biomed. Opt. Express.

[62] Wang TY, Xu C (2020). Three-photon neuronal imaging in deep mouse brain. Optica.

[63] Hontani Y (2022). Deep-tissue three-photon fluorescence microscopy in intact mouse and zebrafish brain. J. Vis. Exp..

[64] Ouzounov DG (2019). GCaMP6 ΔF/F dependence on the excitation wavelength in 3-photon and 2-photon microscopy of mouse brain activity. Biomed. Opt. Express.

[65] Clavreul S (2019). Cortical astrocytes develop in a plastic manner at both clonal and cellular levels. Nat. Commun..

[66] Dray N (2015). Large-scale live imaging of adult neural stem cells in their endogenous niche. Development.

[67] Pedroso GL (2012). Blood collection for biochemical analysis in adult zebrafish. J. Visualized Exp..

[68] Westerfield M (2007). The Zebrafish Book. The Zebra Book. A Guide for the Laboratory Use of Zebrafish (Danio rerio)..

