# Supplementary material for: Label-free imaging of red blood cells and oxygenation with color third-order sum-frequency generation microscopy
Source: Light Sci Appl. 2023 Jan 26;12:29. doi: 10.1038/s41377-022-01064-4 (PMC9879988; doi:10.1038/s41377-022-01064-4)
Supplement: Supplementary file 1 — Supplementary Information [file 41377_2022_1064_MOESM1_ESM.docx]

***Supplementary Information for:***

**Label-free imaging of red blood cells and oxygenation with color third-order sum-frequency generation microscopy**

Júlia Ferrer Ortas^1^, Pierre Mahou^1^, Sophie Escot^1^, Chiara Stringari^1^, Nicolas N. B. David^1^, Laure Bally‑Cuif^2^, Nicolas Dray^2^, Michel Négrerie^1^, Willy Supatto^1^, Emmanuel Beaurepaire^1^*

*^1^ Laboratory for Optics and Biosciences, CNRS, INSERM, École polytechnique, IP Paris, 91128 Palaiseau, France*

*^2^ Zebrafish Neurogenetics Unit, team supported by Ligue Nationale contre le Cancer, Institut Pasteur, UMR3738, CNRS, 75015 Paris, France*

** corresp.* [*emmanuel.beaurepaire@polytechnique.edu*](mailto:emmanuel.beaurepaire@polytechnique.edu)

| **ITEM** | TITLE |
| --- | --- |
| **Suppl. text** | TSFG dependence on pulse duration; effect of chromatic and field aberrations on TSFG |
| **Table T1** | Experimental parameters used for the imaging experiments |
| **Figure S1** | TSFG contrast from point-like objects and horizontal interfaces |
| **Figure S2** | TSFG dependence on pulse duration |
| **Figure S3** | Effect of lateral chromatic aberration on TSFG signals |
| **Figure S4** | 3D THG images of individual fish and human RBCs recorded at successive excitation wavelengths in the 1120-1300 nm range |
| **Figure S5** | THG and TSFG images of human RBCs in different orientations |
| **Figure S6** | Pulses spectral bandwidths and hemoglobin absorption bands |
| **Figure S7** | Specific detection of RBCs among signals from surrounding structures using color TSFG microscopy |
| **Figure S8** | Specific detection of RBCs among signals from pigmented cells and yolk using color TSFG microscopy |
| **Movie M1** | THG spectral imaging of RBCs |
| **Movie M2** | RBC detection in 2 dpf zebrafish embryos |
| **Movie M3** | 3D map of the vascular system in the entire 3 dpf wild type embryo |
| **Movie M4** | Specific detection of RBCs among TSFG signals from other structures in the trunk |
| **Movie M5** | Specific detection of RBCs among TSFG signals from surrounding structures and myelinated axons |
| **Movie M6** | Specific detection of RBCs among nearby pigmented cells |
| **Movie M7** | RBC dynamics in a live adult zebrafish |
| **Movie M8** | Label-free color TSFG images recorded at successive depths in a live adult zebrafish brain |
| **Movie M9** | 3D rendering of an adult zebrafish telencephalon |
| **Movie M10** | 3D rendering of the vasculature in an adult zebrafish telencephalon |

Supplementary Text

Expected TSFG dependence on pulse duration

In this section, we derive the dependence of TSFG signals on pulse duration described in Eqs. 1-2. We denote $E_{1}$, $P_{1}$, $I_{1}$, $\omega_{1}$ and $\tau_{1}$ the field, average power, intensity, frequency and pulse duration of the pump, respectively, and $E_{2}$, $P_{2}$, $I_{2}$, $\omega_{2}$ and $\tau_{2}$ the field, average power, intensity and frequency of the parametric beam, respectively. We assume identical beam waists for the two beams. We assume Gaussian temporal profiles and identical repetition rate 1/T for the two beams:

$$I_{1}\left( t \right)=\left( {I_{0}}/\sqrt{2\pi} \right)\left( T/{\tau_{1}} \right)\exp\left\{ -{t^{2}}/{2{\tau_{1}}^{2}} \right\}$$

The THG field at $3\omega_{1}$ is proportional to $\chi^{(3)}{E_{1}}^{3}$. The TSFG field at $2\omega_{1}+\omega_{2}$ is proportional to ${3\chi}^{(3)}{E_{1}}^{2}E_{2}$. Consequently, the average THG intensity at $3\omega_{1}$ is proportional to $\left\langle{I_{1}}^{3} \right\rangle$ and the average TSFG intensity at $2\omega_{1}+\omega_{2}$ is proportional to $9\left\langle{I_{1}}^{2}I_{2} \right\rangle$.

Let us first express the intensity of a THG signal. The average excitation intensity is:

$$\left\langle I_{1} \right\rangle= \frac{\int_{-T/2}^{+T/2} I_{1}\left( t \right) dt}{T/2-(-T/2)}=I_{0}\frac{T}{\tau_{1}}\frac{1}{T\sqrt{2\pi}}\int_{-T/2}^{+T/2} e^{-\frac{t^{2}}{2\tau_{1}^{2}}} dt=I_{0}$$

We assume that there is no interaction between pulses, so that $\tau_{1}\ll T$ . In this case the integral limits can be extended to $\pm\infty$ . Using the property $\int_{-\infty}^{+\infty} e^{-{\alpha t}^{2}} dt=\sqrt{\pi/\alpha}$ with $\alpha= 1/{2\tau_{1}^{2}}$ , the THG signal at $3\omega_{1}$ is proportional to:

$$S_{THG1}\propto\left\langle{I_{1}}^{3} \right\rangle= \left( \left\langle I_{1} \right\rangle\frac{T}{\tau_{1}} \right)^{3} \frac{1}{\left( \sqrt{2\pi} \right)^{3}}\frac{1}{T} \int_{-T/2}^{+T/2} e^{-\frac{{3t}^{2}}{2\tau_{1}^{2}}} dt= \left( \frac{T}{\tau_{1}} \right)^{2}\left\langle I_{1} \right\rangle^{3}\frac{1}{2\pi\sqrt{3}}$$

Similarly, the TSFG signal at frequency ${2\omega}_{1}+\omega_{2}$ is proportional to:

$$S_{TSFG1}\propto9\left\langle{I_{1}}^{2}I_{2} \right\rangle= 9\left( \left\langle I_{1} \right\rangle\frac{T}{\tau_{1}} \right)^{2} \left( \left\langle I_{2} \right\rangle\frac{T}{\tau_{2}} \right) \frac{1}{\left( \sqrt{2\pi} \right)^{3}}\frac{1}{T} \int_{-T/2}^{+T/2} e^{-(\frac{1}{\tau_{1}^{2}}+\frac{1}{2\tau_{2}^{2}})t^{2}} dt= 9\frac{T^{3}\sqrt{3}}{\tau_{1}\sqrt{\tau_{1}^{2}+2\tau_{2}^{2}}}\left\langle I_{1} \right\rangle^{2}\left\langle I_{2} \right\rangle\frac{1}{2\pi\sqrt{3}}$$

Using analogous calculations for the two other signals we obtain Eqs. 1-2.

Experimental TSFG dependence on pulse duration

In this section, we describe the experimental verification of the TSFG dependence on the pulse duration of the two excitation laser beams predicted by Eqs. 1-2. Since we do not have direct control of these durations, our approach is to compare signals recorded through a TSFG (dual-beam) process with signals recorded through a THG (single beam) process at the same wavelengths (373 and 401 nm).

Assuming〈 P_1_ 〉/〈 P_2_ 〉= 1 for simplicity and setting $\alpha= \tau_{2} / \tau_{1}= \tau_{opo}/\tau_{pump}$, we derive the following relations from Eq. 1:

|  | $\frac{S_{\mathrm{TSF}G_{1}}}{S_{\mathrm{THG}}}= \frac{9\sqrt{3} \alpha}{\sqrt{1/{\alpha^{2}}+2}} \frac{S_{\mathrm{TSF}G_{2}}}{S_{\mathrm{THG}}}= \frac{9\sqrt{3} \alpha}{\sqrt{\alpha^{2}+2}}$ | ( S1 ) |
| --- | --- | --- |

These expressions are plotted in Fig. S2a. TSFG signals are maximized when the pulse duration of the excitation beams are shortest and equal ($\alpha=1).$

Eq. S1 and Fig. S2a consider the general case of a constant $\alpha$ parameter. In reality, however, pulse duration in our OPO system exhibits spectral dependence. To include it in our calculations, we measured the pulse duration after the objective for successive wavelengths spanning the 1045 - 1300 nm spectral range (Fig. S2b). We obtained different values for $\tau_{2}$ at different tuning wavelengths. Taking this into account, the expected TSFG/THG signal ratios can be expressed as:

|  | $\frac{S_{\mathrm{TSF}G_{1}}(373 nm)}{S_{\mathrm{THG}}(373 nm)}= \frac{9\sqrt{3} \alpha''\beta''}{\sqrt{1/{{\alpha'}^{2}}+2}} \frac{S_{\mathrm{TSF}G_{2}}(401 nm)}{S_{\mathrm{THG}}(401 nm)}= \frac{9\sqrt{3} \alpha'''\beta'''}{\sqrt{\alpha^{'2}+2}}$ | ( S2 ) |
| --- | --- | --- |

Here, we have defined $\alpha'= \tau_{2}\left( 1300\mathrm{nm} \right)/ \tau_{1}$, $\alpha''= \tau_{2}\left( 1120\mathrm{nm} \right)/ \tau_{1}$, $\alpha'''= \tau_{2}\left( 1200\mathrm{nm} \right)/ \tau_{1}$, $\beta'' = \tau_{2}\left( 1120\mathrm{nm} \right)/ \tau_{2}\left( 1300\mathrm{nm} \right)$and $\beta''' = \tau_{2}\left( 1200\mathrm{nm} \right)/ \tau_{2}\left( 1300\mathrm{nm} \right)$. These parameters take into account the OPO wavelengths (1120, 1200 and 1300 nm) involved in single-beam THG at 373 nm, single-beam THG at 400 nm and dual-beam TSFG signals at 373 and 401 nm.

The numerical values of the ratios in Eq. S2 calculated from the measured pulse durations are 2.7 and 4.7, respectively.

We then experimentally determined these ratios as follows. For each wavelength (373 and 401 nm), we sequentially acquired TSFG and THG z-stacks of a water-glass interface and computed the TSFG/THG ratio of their z-projection. As presented in Fig. S2c, we obtained pixel value distributions centered at 2.5 ± 0.7 and at 4.3 ± 1.4, respectively, which match well to the values predicted by Eq. S2.

Effect of chromatic and field aberrations on TSFG

We analyzed the effect of chromatic aberration across the field of view on TSFG signals using the approach described in (*1*). We used a preparation of 0.5 µm fluorescent polystyrene beads (TetraSpeck, ThermoFisher Scientific, MA, USA) embedded in an agarose gel and recorded 3D point spread functions with pump (1045 nm) and OPO (1300 nm) excitation on independent detectors. We automatically extracted the axial resolution across the field of view for each beam (Fig. S3a), and the lateral mismatch between the two foci (Fig. 1f and Fig. S3b).

We then estimated the effective field of view for TSFG imaging by recording z-stacks of a horizontal water-glass interface with three simultaneous contrast mechanisms, namely TSFG_1_ at 373 nm, TSFG_2_ at 401 nm, THG_2_ at 433 nm. We projected these stacks along z, and extracted lateral profiles across the field of view. The first graph in Fig. S3c shows the lateral TSFG profiles normalized to the THG profile. A drop of 30±10% in TSFG signal relative to the THG signal is measured at the edges of the field of view. This drop can be caused by several types of field-dependent aberrations causing a reduced overlap of the two excitation beams: (i) a reduced lateral overlap of the two excitation beams can result from lateral chromatic shift away from the optical center (see (*1*)); (ii) axial chromatic shift, e.g. due to different field curvature for the two excitation wavelengths, and (iii) differential degradation of foci sizes, in particular in the axial direction. To analyze the effect of lateral chromatic aberration (i), we used a simple model where the focused excitation beams are described by laterally shifted Gaussian profiles in the imaging plane, termed *pump* and *opo*. In Fig. S3d, we show the calculated integral of triple products TSFG_1_ = $pump^{2}\times opo$, TSFG_2_ = $pump \times opo^{2}$ and THG_2_ = $opo^{3}$ as a function of the spatial mismatch between beams. This simple model suggests that lateral chromatic aberration is a dominant source of TSFG/THG ratio loss near the edges of the field of view. Additional loss may be attributed to the differential change in axial sizes of the two foci far from the optical center, measured in Fig S3a. This differential degradation of PSFs has the effect of changing the effective overlap of the two beams and therefore to alter TSFG signals differently than THG signals. Finally, we generally observed that the difference in field curvature for the two beams was small compared to lateral shifts.

| experiment / figure | lateral pixel size (µm) | axial sampling (µm) | pixel dwell time (µs) | original image size (pixels) | frame rate (Hz) | excitation power (mW) | excitation objective NA |
| --- | --- | --- | --- | --- | --- | --- | --- |
| Fig 2a (right), Fig S4, M1, human | 0.15 | 0.5 | 5 | 320×290 | 0.55 | <20 | 1.05 |
| Fig 2a (right), Fig S4, M1, zebrafish | 0.15 | 0.5 | 5 | 266×262 | 0.08 | <20 | 1.05 |
| Fig 2a (left), Fig S5 | 0.13 | 0.5 | 5 | 498×404 | 0.38 | <20 | 1.05 |
| Fig 2c, M2 | 0.24 | 1 | 5 | 90×120 | 0.81 | <20 | 1.05 |
| Fig 3c, Fig 3d | 0.8 | 0.5 | 5 | 168×890 | 0.85 | <60 | 1.05 |
| Fig 3e, Fig S7, Fig S8, M3-6 | 0.39 | 5 | 6 | 1024×1024 (each tile) | 0.14 | <60 | 1.05 |
| Fig 5b (center), M7 | 0.56 | 0 | 6 | 512×512 | 0.57 | <12 | 1.05 |
| Fig 5b (stack), Fig 5c, M8-10 | 0.35 | 2 | 6 | 1024×1024 | 0.14 | ≤135 | 1.05 |
| Fig S1 | 0.35 | 0.1 | 5 | 326×230 | 0.29 | <30 | 1.05 |

**Table T1. Experimental parameters used for the imaging experiments.**


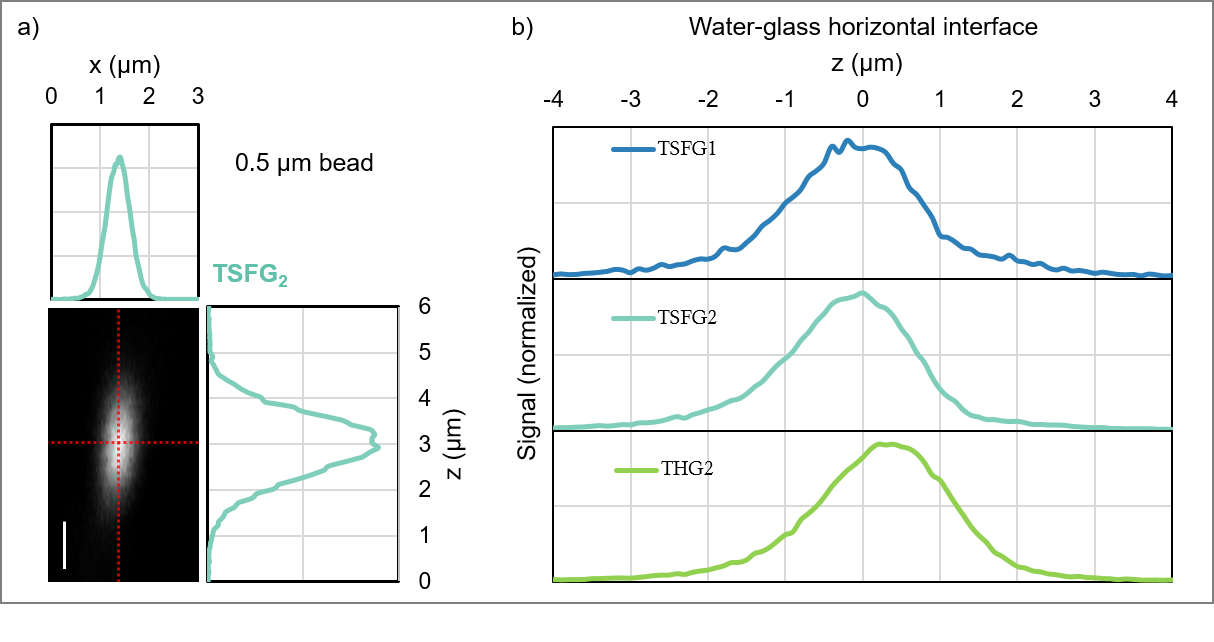


Fig. S1. TSFG contrast from point-like objects and horizontal interfaces. (a) XZ projection of a 3D image of a 0.5 μm diameter polystyrene bead obtained with TSFG contrast (detection at 401nm). The lateral resolution estimated from N=7 different beads is 0.51±0.03 μm FWHM. The axial resolution estimated on the same beads is 1.79±0.03 μm FWHM. Similar values were found for TSFG1 and THG2 signals. Note that these values are an overestimate of the lateral resolution, given the diameter of the beads. (b) TSFG and THG axial profiles acquired simultaneously across a horizontal water-glass interface. All signals exhibit a peak when the interface is in focus and no signal when the focus is within a homogeneous region (water or glass), indicating that the phase matching conditions are similar for THG and TSFG under our experimental conditions.


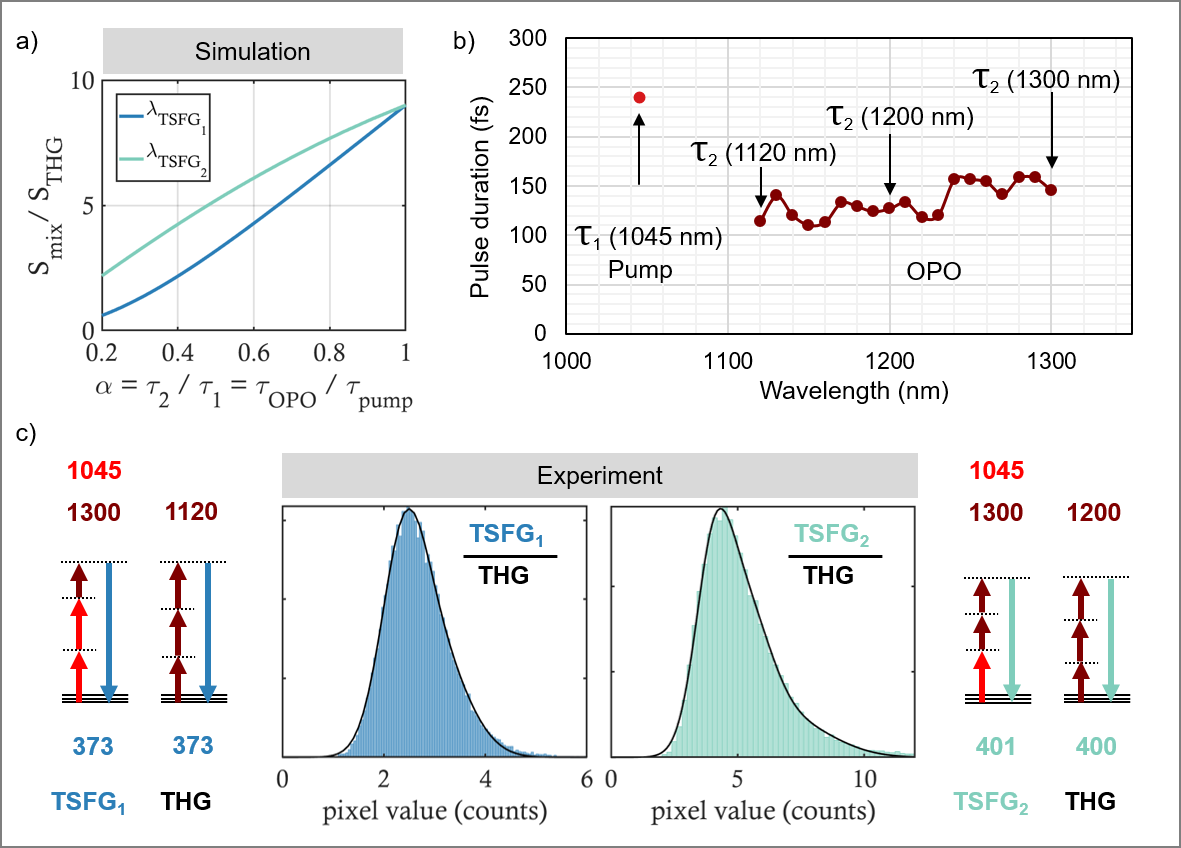


Fig. S2. TSFG dependence on pulse duration. (a) Representation of S_TSFG_/S_THG_ in terms of the parameter $\boldsymbol{\alpha=}\boldsymbol{\tau}_{\boldsymbol{2}}\boldsymbol{/}\boldsymbol{\tau}_{\boldsymbol{1}}$ in the case of no spectral dependence of the OPO beam pulse duration, as described by Eq. S1. (b) Experimental autocorrelation measurements of the pulse durations of the pump and OPO beams at the sample plane after dispersion compensation to achieve the shortest durations at each wavelength. A Gaussian temporal profile was assumed. (c) Pixel value distribution of the image resulting from the ratio of a TSFG (dual beam) image and a THG (single beam) image acquired at the same wavelength. The energy schemes of the two excitation modes for the two analyzed wavelengths are shown on each side of the histograms. The distributions are centered at 2.5 ± 0.7 and 4.3 ± 1.4 for 373 and 401 nm, respectively.


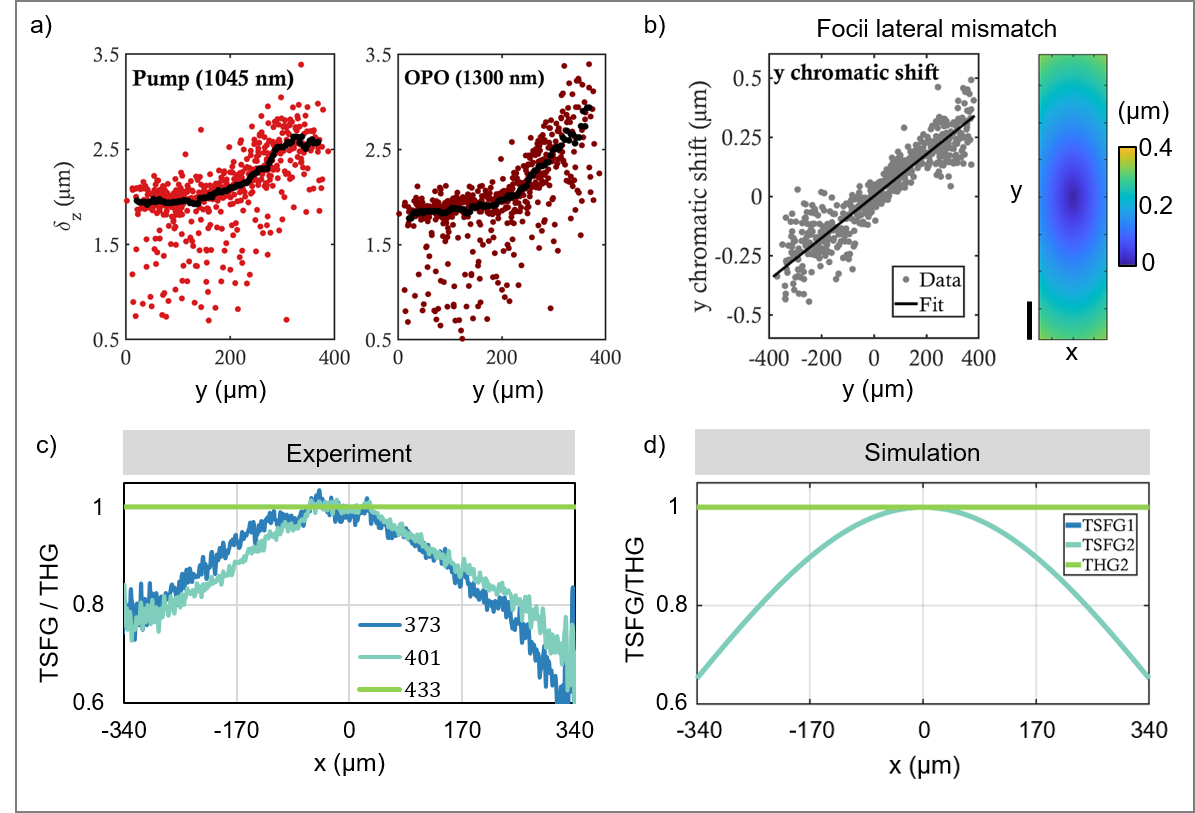


Fig. S3. Effect of lateral chromatic aberration on TSFG/THG signal ratio. (a) Axial resolution as a function of the distance from the center of the field of view for the pump and OPO beams, measured using the procedure described in *(1)*. Note that ratio between the axial sizes of the two foci changes as a function of distance from the optical center, as a result of axial chromatic aberration. (b) Lateral chromatic shift between the two beams along the y dimension. Scale bar: 100 μm. Similar values were found for the x direction. (c) TSFG signal profiles measured on a water-glass interface along the field of view normalized to the single-beam THG profile. The TSFG/THG signal ratio decrease by approximately 30% 350 µm away from the center of the field of view. (d) Simple simulation of the TSFG signal from a point-like object across the field of view, assuming the lateral mismatch evaluated in (b), namely 85 nm every 100 µm. Both excitation beams are considered to have a Gaussian profile with 0.3 µm width at 1/e^2^. The calculation suggests that lateral chromatic shift accounts for most of the drop in the TSFG/THG ratio observed away from the optical center. An additional drop of limited magnitude is expected due to the differential changes in axial sizes of the two foci far from the optical center (a).


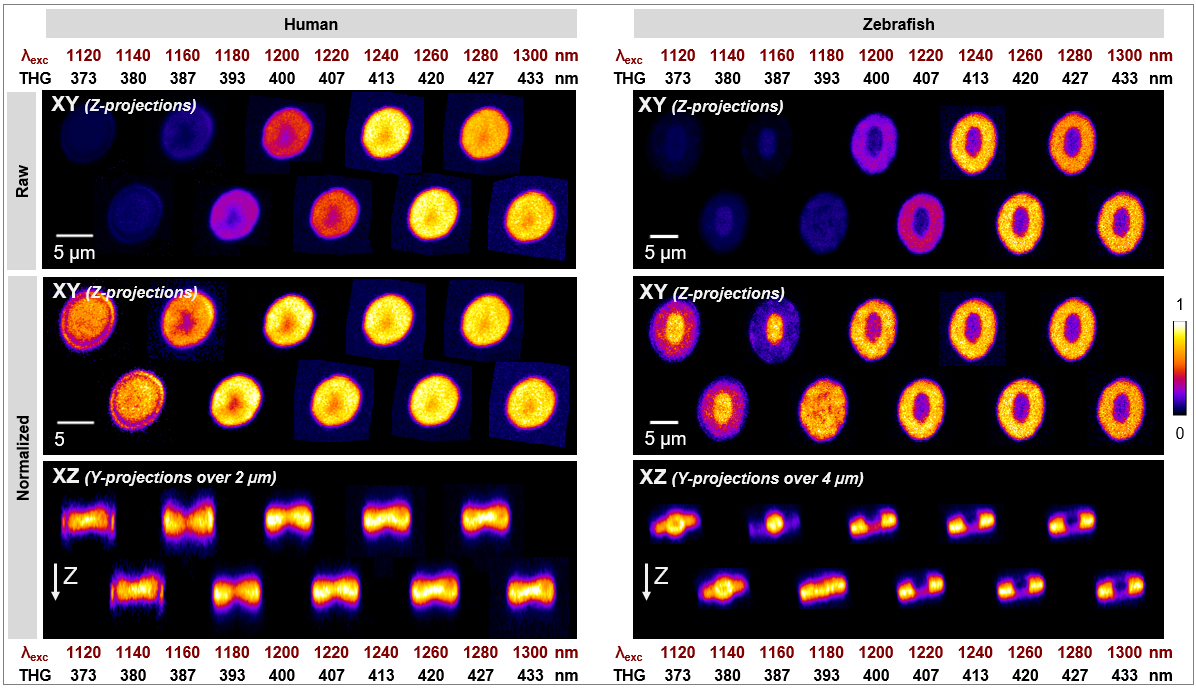


Fig. S4. 3D THG images of individual fish and human RBCs recorded at successive excitation wavelengths in the 1120-1300 nm range. 3D images of individual RBCs were recorded at successive excitation wavelengths in the 1120-1300 nm range and normalized by the excitation power and pulse duration. A 10-20× signal enhancement is observed when the emission wavelength matches the Soret band, attributed to three-photon resonance. The images are normalized to their maxima except in the first line to illustrate the signal enhancement. XY images are Z-projections over the entire imaged volume. XZ images are Y-projections of the central planes over a thickness 2 µm for the Human RBC and 4 µm for the zebrafish RBC.


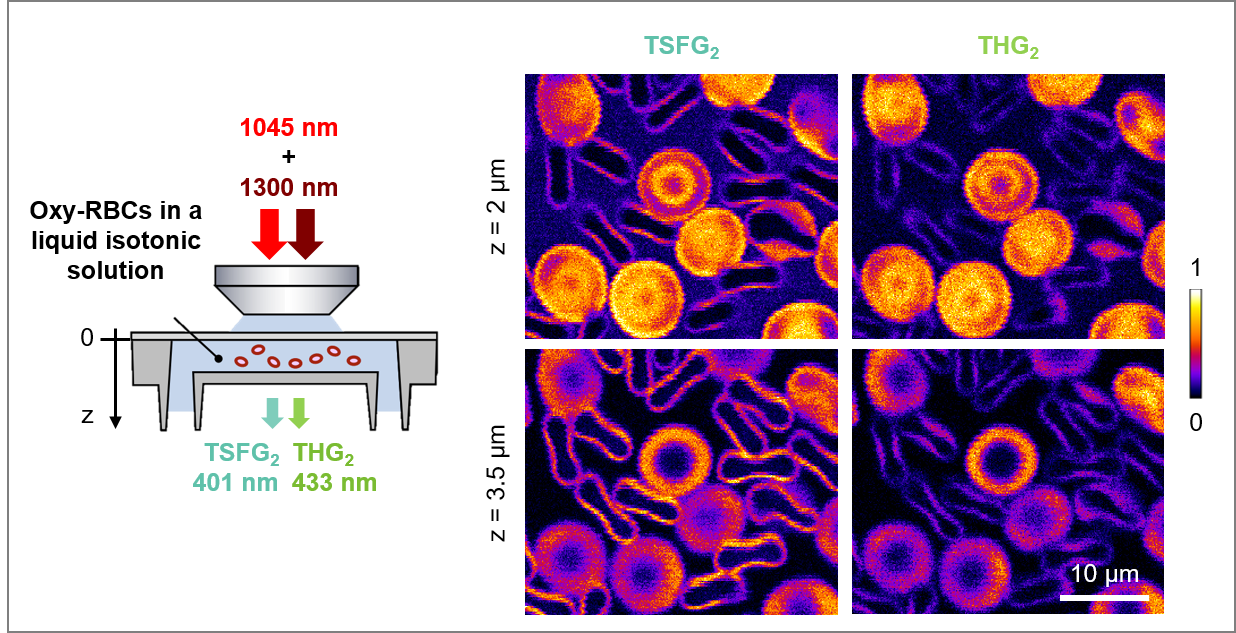


Fig. S5. THG and TSFG images of human RBCs in different orientations. 2D images of a group of RBCs in oxygenated state were recorded at different depths. The data illustrate that THG and TSFG images highlight interfaces.


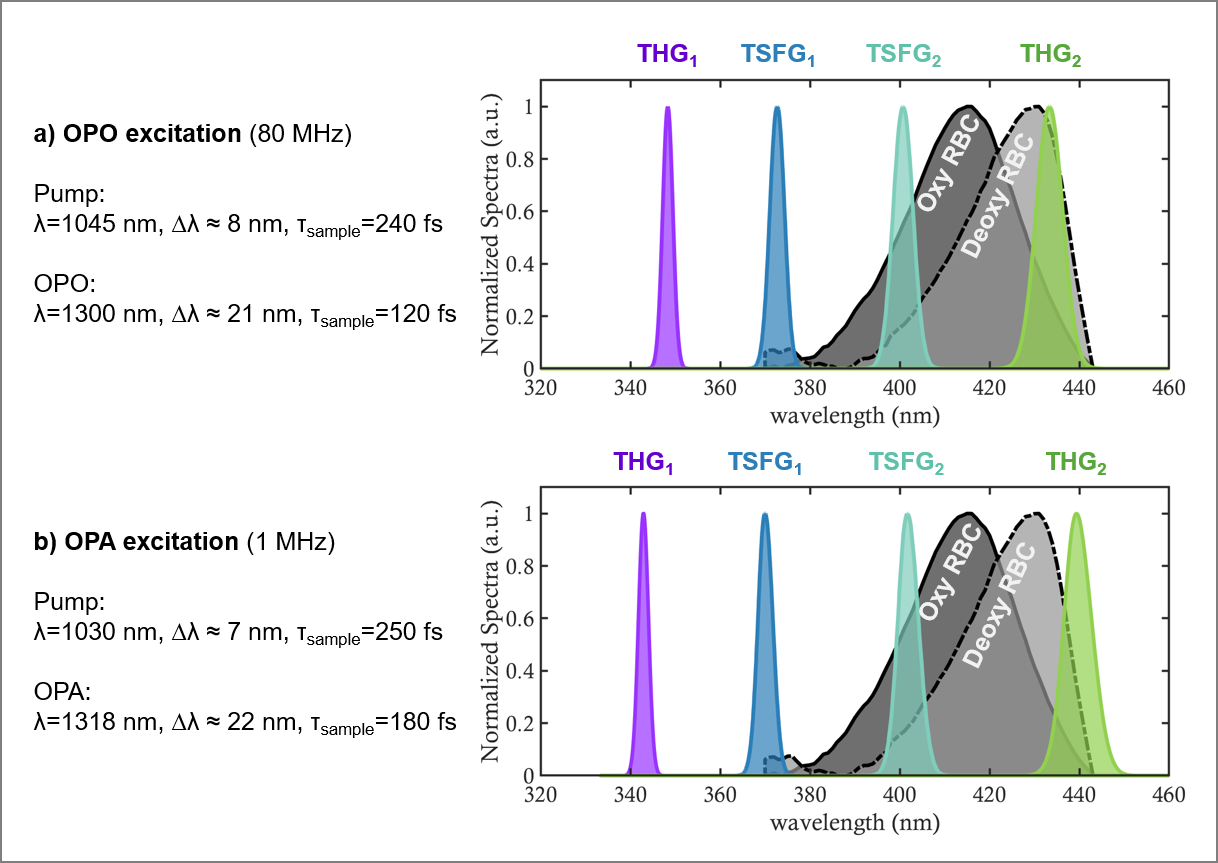


Fig. S6. THG-TSFG spectral bandwidths and hemoglobin absorption bands. THG and TSFG spectral bandwidths are estimated based on the spectral bandwidth of the excitation pulses of (a) the 80 MHz OPO system and (b) the 1MHz OPA system used in this study. It is interesting to note that hemoglobin has broad absorption bands in the Soret region, making it possible to use femtosecond pulses to enhance third-order signals while preserving specificity.


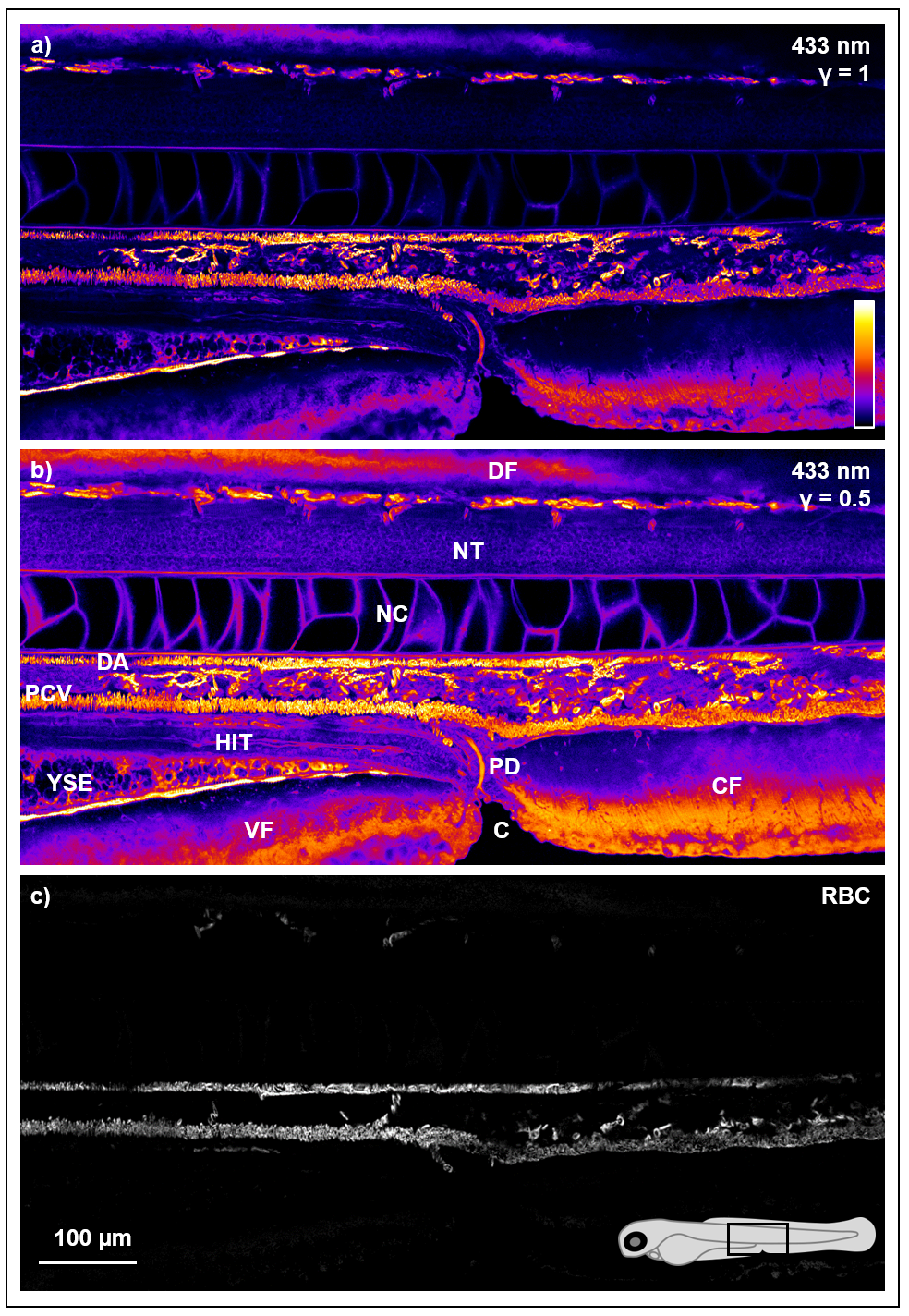


Fig. S7. Specific detection of RBCs among signals from surrounding structures using color TSFG microscopy. Selected image from the mosaic acquisition presented in Fig. 3e and Movies S3-4 of a wild type 3 dpf entire embryo. A gamma compression (γ = 0.5) was applied to the pixel intensity values of the raw THG image at 433 nm (a) to reveal all detected structures in the trunk using THG signals (b), such as the dorsal fin (DF), the caudal fin (CF), the ventral fin (VF), the neural tube (NT), the notochord (NC), the dorsal aorta (DA), the posterior cardinal vein (PCV), hindgut intestinal track (HIT), the pronephric duct (PD), yolk sac extension (YSE) and the cloaca (C). Specific RBC segmentation is obtained from color TSFG signals (c). Black box indicates where the image was acquired within the embryo.


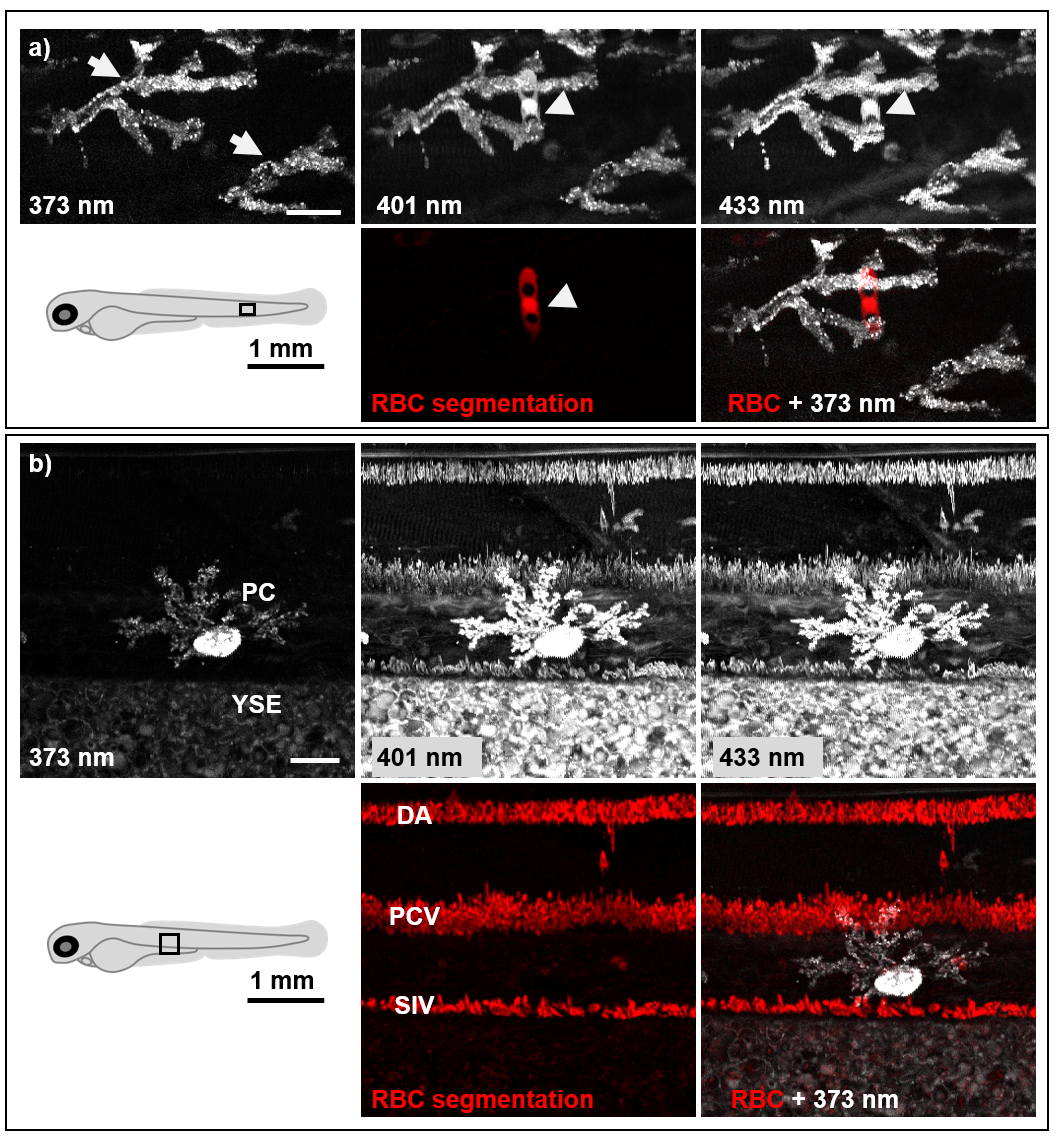


Fig. S8. Specific detection of RBCs among signals from pigmented cells and yolk using color TSFG microscopy. Color TSFG images detected at 373, 401 and 433 nm are used to generate RBC segmentation in red (see Methods). (a) a RBC (arrow heads) is shown among xanthophore pigmented cells (arrows) in a 3 dpf *casper* embryo. 15 µm thick z-projection. See also Movie M6. (b) Blood vessels (DA: dorsal aorta; PCV: posterior cardinal vein; SIV: subintestinal vein) of a 3 dpf wild type embryo are detected in a region comprehending different structures such as the yolk sac extension (YSE) or a pigmented cell (PC). 100 µm thick z-projection. Black boxes indicate where the image was acquired within the embryo. Scale bar: 20 μm.


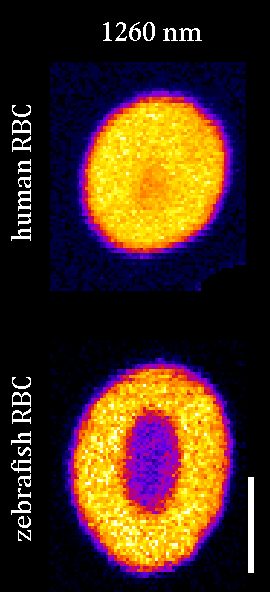


**Movie M1. THG spectral imaging of RBCs.** THG images of human and zebrafish RBCs obtained at different excitation wavelengths. The images were normalized to take into account pulse duration and excitation intensity. They illustrate the 10-20× increase in THG signal near the Soret band due to 3-photon resonant enhancement. Scale bar: 5 μm.


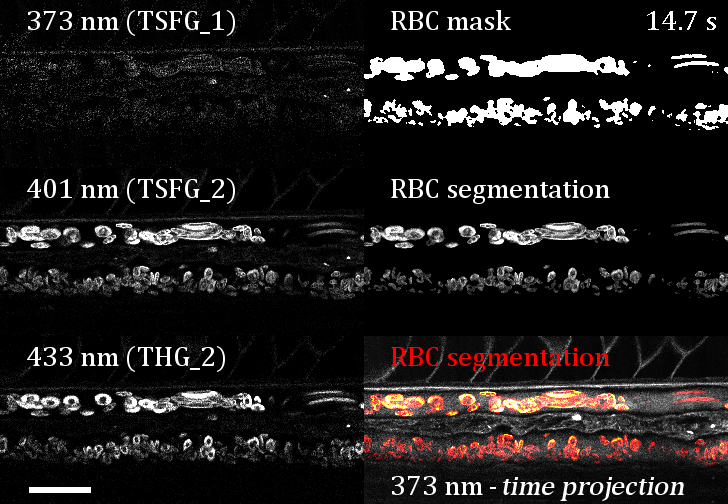


**Movie M2. RBC detection in 2 dpf zebrafish embryos.** (Left) Simultaneously acquired TSFG-THG images of RBCs flowing through the dorsal aorta and posterior cardinal vein. (Right) Image processing to segment RBCs. The RBC mask was obtained as explained in Fig. 3b. On the bottom image, the segmented RBCs were overlaid to the time projection of the TSFG_1_ image at 373 nm (non-resonant signal) providing a structural landmark. Scale bar: 50 μm.

**
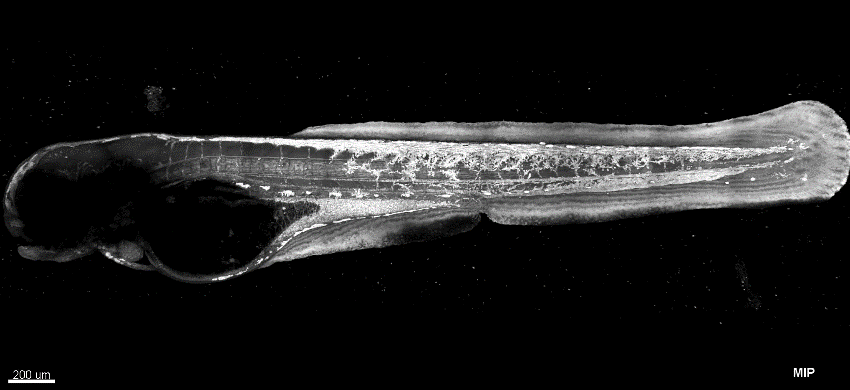
**

**Movie M3. 3D map of the vascular system in the entire 3 dpf wild type embryo.** Zoom in on a maximum intensity projection (MIP) of the TSFG image at 401 nm. 2D view of the central plane (z = 65 μm) **o**f this signal moving along the anterio-posterior axis. 3D rendering of the RBC segmentation using an Enhancement image (see Methods) superimposed to the MIP of the image at 401 nm.


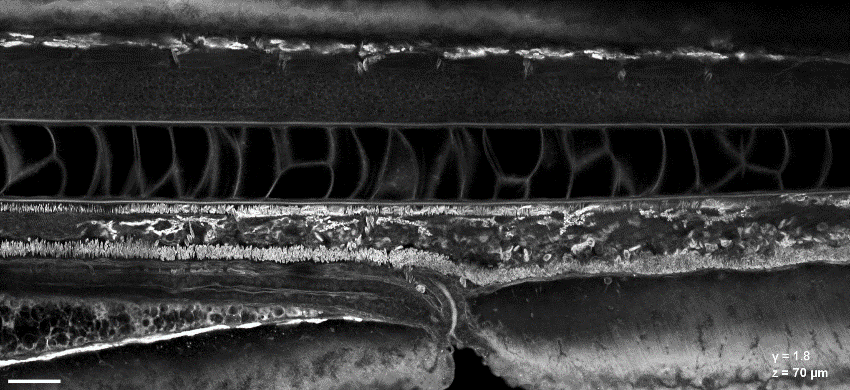


**Movie M4. Specific detection of RBCs among TSFG signals from other structures in the trunk.** Detail of a 3 dpf wild type embryo (shown in Fig. 3e and Movie M3). THG signal at 433 nm together with RBC detection using an Enhancement image (see Methods). Scale bar: 100 μm.


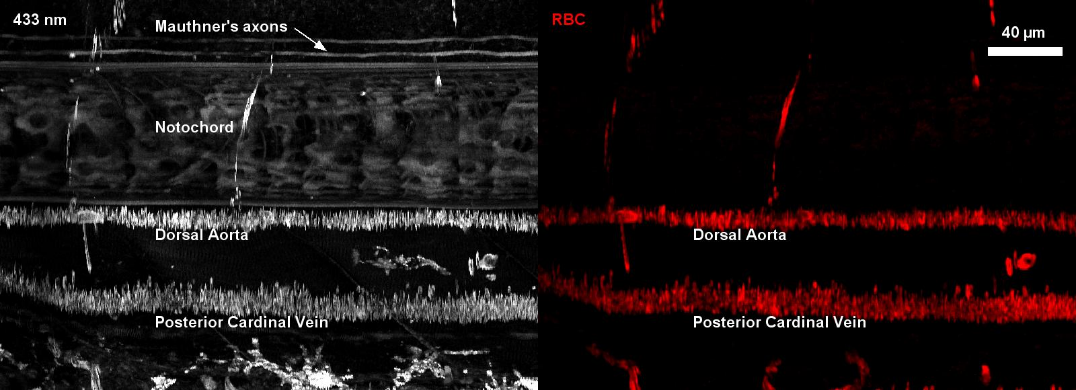


**Movie M5. Specific detection of RBCs among TSFG signals from surrounding structures and myelinated axons.** THG signal at 433 nm in a 3dpf *casper* zebrafish embryo including a strong signal from myelinated Mauthner's axons together with RBC detection using an Enhancement image in red (see Methods).


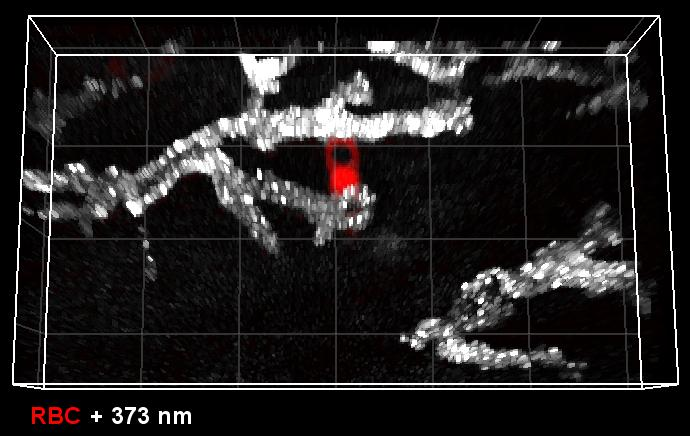


**Movie M6. Specific detection of RBCs among nearby pigmented cells.** Color TSFG images in a 3dpf *casper* zebrafish embryo including pigmented cells and RBC**s** segmentation using an Enhancement image in red. Grid: **2**0 μm.


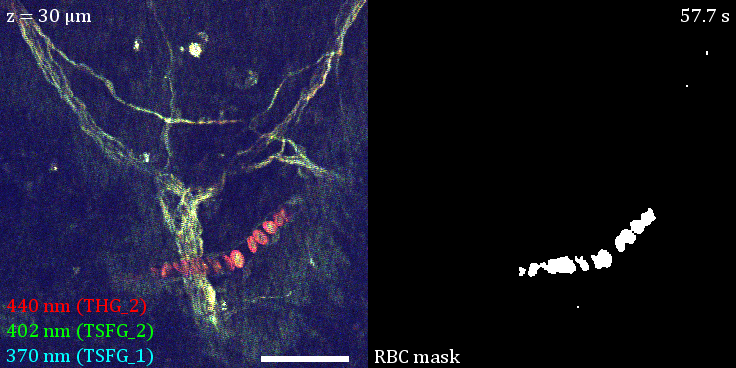


**Movie M7. RBC dynamics in a live adult zebrafish.** (Left) Single flowing RBCs are detected on a blood vessel above the skull (z = 30 μm) close to myelinated axons. (Right) RBCs segmentation using an Enhancement image (see Methods). Scale bar: 50 μm.

**
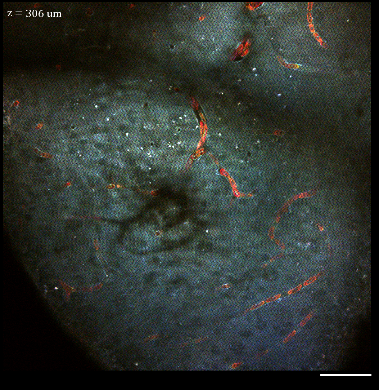
**

**Movie M8. Label-free color TSFG images recorded at successive depths in a live adult zebrafish brain.** Color TSFG imaging reveal blood vessels in the zebrafish telencephalon. Scale bar: 50 μm.

**
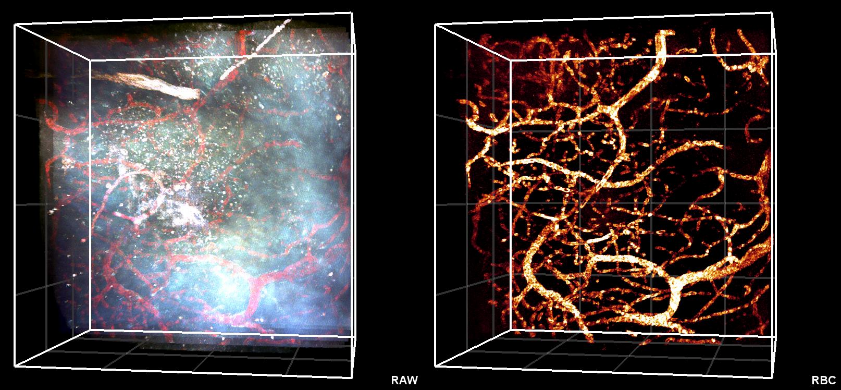
**

**Movie M9. 3D rendering of an adult zebrafish telencephalon.** (Left) Color TSFG images. (Right) RBCs segmented using resonance-enhanced TSFG signals (see Methods). Grid: 50 μm.

**
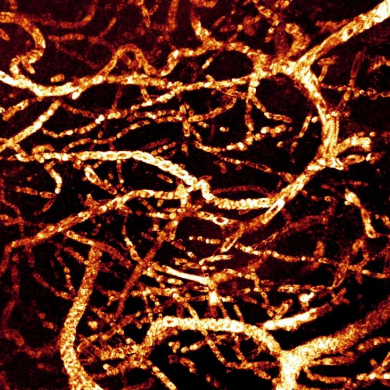
**

**Movie M10. 3D rendering of the vasculature in an adult zebrafish telencephalon.** RBCs were segmented using the resonance-enhanced TSFG signals (see Methods). Size of the field of view: 369 µm x 379 µm x 276 µm.

**References**

1. Mahou, P. *et al*. Metrology of multiphoton microscopes using second harmonic generation nanoprobes. *Small* **13**, 1701442 (2017).
